# Adipocyte–Tumor Interactions in the Bone Marrow Niche: Implications for Metastasis and Therapy

**DOI:** 10.3390/ijms26199781

**Published:** 2025-10-08

**Authors:** Alhomam Dabaliz, Mohammad Nawar Al Hakawati, Najmuddeen Alrashdan, Sarah Alrashdan, Mohamad Bakir, Khalid S. Mohammad

**Affiliations:** 1 Department of Clinical Skills, College of Medicine, Alfaisal University, Riyadh 11533, Saudi Arabia; almdabaliz@alfaisal.edu; 2 Hematogenix, Tinley Park, IL 60487, USA; dr.nawar.pathology@gmail.com; 3 Department of Anatomy, College of Medicine, Alfaisal University, Riyadh 11533, Saudi Arabia; nalrashdan@alfaisal.edu (N.A.); salrashdan@alfaisal.edu (S.A.); 4 Department of Medicine, College of Medicine, Alfaisal University, Riyadh 11533, Saudi Arabia; mbakir@alfaisal.edu

**Keywords:** bone marrow adipocytes, bone metastasis, tumor microenvironment, adipokines, therapeutic resistance, extracellular vesicles

## Abstract

Bone metastases continue to be a major cause of morbidity and mortality in patients with advanced cancers, driven by the dynamic remodeling of the bone marrow niche. Traditionally viewed as passive space-fillers, bone marrow adipocytes (BMAs) are now recognized as active regulators of tumor growth, therapeutic resistance, and skeletal pathology. BMAs comprise a significant portion of the adult marrow space, particularly in aging and obesity, and facilitate metastatic colonization through various mechanisms. These include metabolic coupling, where adipocyte-derived fatty acids fuel tumor oxidative phosphorylation; the secretion of adipokines such as leptin and IL-6, which promote epithelial-to-mesenchymal transition, invasion, and immune evasion; regulation of osteoclastogenesis via RANKL expression; and the release of extracellular vesicles that reprogram cancer cell metabolism. Clinical and experimental studies show that BMA expansion correlates with increased tumor burden and poorer outcomes in breast, prostate, lung cancers, and multiple myeloma. Additionally, BMAs actively promote therapeutic resistance through metabolic rewiring and drug sequestration. Experimental models, ranging from in vitro co-cultures to in vivo patient-derived xenografts, demonstrate the complex roles of BMAs and also reveal important translational gaps. Despite promising preclinical approaches such as metabolic inhibitors, PPARγ modulation, adipokine blockade, and lifestyle changes, no therapies directly targeting BMAs have yet reached clinical practice. This review compiles current evidence on the biology of BMAs, their tumor-promoting interactions, and potential therapeutic strategies, while also highlighting unresolved questions about BMA heterogeneity, lipid flux, and immunometabolic crosstalk. By revealing how bone marrow adipocytes actively shape the metastatic niche through metabolic, endocrine, and immunological pathways, this review highlights their potential as novel biomarkers and therapeutic targets for improving the management of bone metastases.

## 1. Introduction

Bone metastases remain one of the most serious complications of advanced malignancies, frequently occurring in breast, prostate, and lung cancers, as well as in multiple myeloma (MM) [1]. The skeletal system is not only a frequent site of tumor dissemination but also a microenvironment that fosters malignant progression, resulting in substantial morbidity, reduced quality of life, and shortened survival [1]. These outcomes arise largely from the interplay between tumor cells and the bone marrow microenvironment (BMME) [2], where cellular and molecular crosstalk generates a tumor-supportive niche. This microenvironment is composed of osteoblasts, osteoclasts, endothelial and immune cells, mesenchymal stromal cells (MSCs), and bone marrow adipocytes (BMAs), all of which contribute to the pathogenesis of bone metastasis [2,3,4,5]. The high mortality associated with bone-metastatic disease is partly driven by dynamic remodeling of the skeletal microenvironment, which sustains a tumor-supportive niche and therapy resistance. This “vicious cycle” involves reciprocal signaling between tumor cells and bone-resident cells in which tumor-derived factors stimulate bone turnover, releasing growth factors that, in turn, fuel tumor proliferation [1].

While the clinical manifestations differ by tumor type, a unifying theme is that skeletal involvement dramatically worsens prognosis. In prostate cancer, bone metastases are typically osteoblastic, yet paradoxically result in fragile, structurally unsound bones. One explanation for this is the tumor-associated endothelial cells’ ability to transdifferentiate into osteoblasts under the influence of cancer-secreted bone morphogenetic protein 4 (BMP4) [2,3]. In a patient-derived xenograft model producing osteoblastic lesions, endothelial-specific deletion of the osteoblast transcription factor Osterix (Sp7) significantly reduced tumor-induced bone formation [4]. Furthermore, osteoblasts within human prostate cancer bone metastases were found to co-express endothelial markers. By generating new osteoblastic bone within metastatic lesions, this endothelial-to-osteoblast conversion perpetuates the tumor-supportive niche, amplifies skeletal pathology, and reinforces the vicious cycle that underpins the poor prognosis associated with bone metastases [1,2,3,4,5].

By contrast, bone metastases in breast and lung cancers are predominantly osteolytic, driven by excessive osteoclast activity and resulting in pathologic fractures, spinal cord compression, and hypercalcemia. The development of bone metastases in these cancers marks a turning point toward high morbidity and reduced survival, largely due to the burden of skeletal-related events (SREs) [6]. More than half of patients with breast or lung cancer experienced at least one SRE at or after their bone metastasis diagnosis, with cumulative incidence rates at 24 months reaching 54.2% for breast cancer and 47.7% for lung cancer. These events, pathologic fractures, spinal cord compression, and the need for surgery or radiotherapy to the bone, worsen ECOG performance status by causing severe pain and mobility impairment, thereby degrading quality of life [7].

In multiple myeloma, skeletal involvement is a defining feature: ~70–80% of patients have osteolytic lesions at diagnosis, and most patients develop clinically significant bone disease over the disease course despite therapeutic advances [8,9,10,11]. Bone marrow adipocytes (BMAs) are active contributors to the progression and treatment resistance of multiple myeloma. Mature BMAs enhance myeloma cell viability and protect them from chemotherapy-induced apoptosis, in part by triggering autophagy pathways [12,13]. This protective effect was mediated by IL-6 and leptin; while anti-tumor adipokines are often suppressed in the myeloma marrow environment [14].

Systematically reviewed preclinical and clinical studies found that BMAs influence cancer cells by releasing fatty acids, adipokines, and inflammatory cytokines including IL-1β and IL-6 and upregulating fatty acid-binding protein 4 (FABP4), thereby fueling tumor growth and reinforcing a pro-inflammatory phenotype in the marrow niche [14]. Additional studies confirm that BMAs secrete key adipokines like leptin, adiponectin, IL-6, and tumor necrosis factor alpha (TNF-α), each of which can modulate tumor cell behavior. Leptin and IL-6 often support tumor survival and proliferation, whereas adiponectin may have suppressive effects [12,14,15,16]. Extracellular vesicles (EVs), though less studied specifically from BMAs, are known to mediate intercellular communication in adipose-rich environments, transferring lipids, adipokines, and microRNAs (miR), and are implicated in promoting tumor plasticity and metastasis [17,18,19].

BMAs are abundant in the adult skeleton, occupying up to 70% of marrow volume in some regions, and their prevalence increases with multiple physiological and pathological conditions [14]. It is important to note that BMA content expands progressively with aging, particularly in constitutive BMAT-rich distal skeletal sites, and is further augmented by metabolic disorders such as obesity and type 2 diabetes, where altered systemic lipid and glucose metabolism promote adipogenic MSC commitment at the expense of osteoblastogenesis [20]. BMAs engage in dynamic crosstalk with tumor cells, notably in prostate, breast, and melanoma metastases, through the secretion of fatty acids, cytokines, adipokines, and other soluble factors. Their release not only fuels tumor metabolic adaptation and growth but also induces a pro-inflammatory phenotype in BMAs themselves, constituting a feedback loop that reshapes the marrow niche in favor of malignancy [14,21,22,23].

Traditionally considered inert space-fillers, BMAs are now recognized as dynamic regulators of the bone marrow microenvironment. Far from being passive, BMAs actively influence marrow physiology through the secretion of adipokines, cytokines, lipids, and extracellular vesicles. BMAs influence both bone remodeling and hematopoiesis, in part via adipokines such as leptin and adiponectin, and inflammatory mediators IL-6 and TNF-α, which impact marrow stromal and immune compartments [20,24]. BMAs directly participate in tumor–adipocyte crosstalk, supplying fatty acids through FABP4- and peroxisome proliferator-activator receptor gamma (PPARγ)-mediated pathways, while simultaneously releasing IL-1β and TNF-α to establish a pro-tumor inflammatory milieu [14].

At a mechanistic level, BMAs are metabolically flexible, adjusting lipid storage and secretory profiles in response to metabolic and oncogenic stress. This adaptability enables them to act as a reservoir of nutrients for tumor cells while also amplifying signaling pathways that promote proliferation, invasion, and drug resistance [12,25]. BMAs have an active participation in marrow biology, a significant role in regulating vascularity, hematopoietic stem cell activity, and skeletal homeostasis, and ultimately shaping the structural and functional landscape in which malignant cells evolve [17,26].

Despite significant advances in our understanding of BMAs as active components of the bone marrow microenvironment, including their endocrine activity, metabolic regulation of hematopoiesis, and crosstalk with tumor cells, critical knowledge gaps remain. These include the need for human-based studies to validate findings from murine models, clarification of how BMA-secreted factors influence different metastatic lineages, insight into how their secretome evolves during therapy, and a more complete understanding of how BMAs integrate into the broader marrow ecosystem [14,19]. These gaps are critical, as BMAs not only represent a large and dynamic cell population in adult marrow but also one that is profoundly altered by aging and therapy, the very contexts in which cancer patients develop bone metastases.

The aim of this review is therefore to synthesize current evidence on the role of bone marrow adipocytes in bone metastases, with a particular focus on their contribution to tumor metabolism, cytokine signaling, and marrow niche remodeling. By examining their biological origins, expansion under physiological and pathological conditions, and mechanistic roles in cancer progression, we seek to highlight BMAs as active stromal regulators rather than passive bystanders, and to identify critical research gaps that must be addressed to define their potential as therapeutic targets in metastatic disease fully.

## 2. Biology of Bone Marrow Adipocytes

### 2.1. Origin and Differentiation

BMAs originate from mesenchymal stromal cells (MSCs), a multipotent population capable of differentiating into osteoblasts, chondrocytes, and adipocytes. Lineage commitment toward the adipocyte fate is governed by a coordinated transcriptional program, with PPARγ and CCAAT/enhancer-binding protein alpha (C/EBPα) acting as master regulators of adipogenesis. Activation of the PPARγ–C/EBPα axis drives the expression of adipocyte-specific genes, promotes lipid droplet formation, and suppresses osteogenic differentiation pathways, thereby shifting the MSC fate balance toward marrow fat deposition [24]. There are two spatially and functionally distinct subtypes of marrow adipose tissue (MAT): constitutive MAT (cMAT) and regulated MAT (rMAT). Constitutive MAT develops early in life, is relatively stable, and resides predominantly in the distal skeleton, whereas regulated MAT is more metabolically dynamic, located in the proximal skeleton, and responsive to systemic and local cues such as dietary changes, endocrine status, and hematopoietic demand [24].

### 2.2. Spatial Heterogeneity

Bone marrow adipose tissue (BMAT) is regionally heterogeneous: constitutive BMAT predominates in distal appendicular sites and is relatively stable, whereas regulated BMAT is enriched at axial/proximal locations and is metabolically responsive to systemic cues (diet, endocrine status, hematopoietic demand) [24]. Human imaging data support this site-specific behavior: in adolescents and young adults with obesity, distal tibial (appendicular) MAT correlates inversely with local trabecular volumetric bone mineral density (vBMD) and microarchitecture, whereas lumbar spine (axial) MAT showed no such relationship to distal tibial bone parameters, underscoring that associations between MAT and bone depend on the site [27]. In cancer research, reviews suggest that BMAT in metabolically dynamic (often axial) niches may foster tumor–stroma crosstalk via lipids and cytokines, but much of this comes from murine work, with scant high-resolution human evidence [14]. Emerging single-cell and spatial multi-omics methodologies can map depot-specific BMA states and their interactions in situ, yet current platforms face practical constraints (tissue handling, integration of modalities) and have been sparingly applied to human BMAT, highlighting a clear translational gap ripe for focused studies in human axial vs. appendicular marrow [28].

## 3. Mechanistic Crosstalk Between Bone Marrow Adipocytes and Tumor Cells

Multiple studies have explored the relationship between adipocytes and tumor growth [29,30,31]. Many tumors promote different activities within adipocytes through the expression of various factors, and vice versa [32,33,34]. While this applies to all adipocytes in the body, BMAs in particular play a major role in bone metastasis.

BMAs are the most abundant cell type in the bone marrow, and a major regulator of bone homeostasis [35]. These cells interact with tumor cells in many ways that ultimately give them a significant role in the development of bone metastasis [36]. Bone marrow adipocytes influence bone metastasis through multiple interconnected mechanisms that span metabolic, endocrine, immunologic, and skeletal pathways.

### 3.1. Metabolic Coupling

Tumor cells influence BMA metabolism in many ways. One of the major metabolic shifts they promote in BMAs is a catabolic shift towards lipolysis [31,37]. This is achieved by the secretion of certain factors like parathyroid hormone-related peptide (PTHrP) or by the phosphorylation of hormone-sensitive lipase (HSL) and perlipin A, the rate-limiting enzyme for lipolysis and the gatekeeper of lipid droplets, respectively, all of which ultimately inhibit adipogenesis and induce lipolysis in adipocytes [38,39,40,41,42]. The free fatty acids generated from lipolysis are then transported to the surrounding metastasized tumor cells to assist in their growth [43]. It is theorized that tumor cells promote such activity because they act as “metabolic parasites” that harvest their metabolic needs from surrounding cells, such as adipocytes [37]. Metastasized tumor cells also promote the expression of CD36 on adipocytes [44]. This membrane glycoprotein is involved in importing adipocyte-released fatty acids into cancer cells [45]. High expression of CD36 is associated with the expression of various molecules responsible for fatty acid transport and metabolism [44]. Among these molecules is FABP4, which acts as a chaperone that shuttles fatty acids along subcellular locations [44,46,47]. These two molecules appear to play a crucial role in cancer cell survival, as their chemical inhibition in one study led to the apoptosis of cancer cells [44]. BMAs can also influence the metabolism in tumor cells that metastasize to bone. A notable example of this is the promotion of oxidative phosphorylation in these tumor cells. Most tumor cells demonstrate the Warburg phenotype, which relies on aerobic glycolysis for energy generation and shunts the produced pyruvate to lactate [48]. However, it is notable that certain cancers, such as multiple myeloma and leukemias, can utilize oxidative phosphorylation effectively for energy generation [49]. A proposed mechanism that may explain this phenomenon is the increased production of acetyl-CoA resulting from fatty acid oxidation by nearby adipocytes. These cancer cells promote the breakdown of lipids, as discussed earlier, which feeds the mitochondria of these tumor cells with the necessary materials for the TCA cycle and oxidative phosphorylation [50].

### 3.2. Adipokine Signaling

Adipose tissue can be considered the largest endocrine organ in the body, secreting factors like adipokines and cytokines [51]. These factors represent a crucial medium for the interaction between adipocytes and the metastasized tumor cells. Leptin, an adipokine secreted by adipocytes, has been shown to affect bones in many ways. It can bind to different components of the bone by the Ob receptor (Ob-R) to modulate bone density and promote BMA differentiation of bone marrow mesenchymal stem cells [52]. In addition to its effect on the bone, leptin has been shown to be involved in the promotion of growth and metastasis of cancers like breast cancer and multiple myeloma [53,54,55,56]. These studies reveal that tumor cell expression of leptin, as well as Ob-R correlates with aggressive tumor behavior [57]. Among the ways leptin accelerates metastasis is by promoting the epithelial to mesenchymal transition (EMT) [58], inducing tissue metalloprotease (MMP) secretion [59], influencing macrophages to become tumor-associated macrophages that support tumor growth and metastasis [60], inducing bone erosion by enhancing the expression of soluble intracellular adhesion molecule (sICAM)-1, which promotes osteoclastogenesis [61], and promoting PLOD2 expression in metastasized cells, an enzyme that can be used for collagen reorganization to better assist in cancer cell migration and invasion [62,63]. IL-6 is another major cytokine produced by adipocytes [64]. This factor binds to IL-6 receptors in the tumor microenvironment, leading to many effects like the induction of EMT and the upregulation of E-cadherin and MMP via the activation of the Jak2-STAT3 signaling pathway [65,66], the promotion of cancer cell survival by the activation of the PI3K/Akt pathway [64], as well as promote PLOD2 expression in cancer cells alongside leptin [62,63]. While BMAs abundantly release IL-6, their release of TNF-α is much more subtle [67]. However, some cancers, like multiple myeloma, produce TNF-α to suppress the secretion of adiponectin, [68] an adipokine with significant antitumor effects [69,70], from adipocytes. This effect is especially pronounced on BMAs as they are one of the major producers of adiponectin in the body [53]. BMA’s production of IL-1β is another driver for metastasis. This effect was primarily observed in breast cancer metastasis, where this cytokine interacts with endothelial cells by increasing VEGF receptors on them and activating the p38-MAPK signaling pathway, leading to endothelial cell migration, tube formation, increased capillary permeability, and vasculogenesis [71,72]. All these effects of adipokines give insight into why the presence of BMAs is a major contributor to a cancer’s ability to metastasize to the bone.

### 3.3. Immunologic Crosstalk

Adipocyte-derived IL-6, IL-1β and chemokines drive MDSC recruitment and TAM polarization, blunting B- and T-cell function. BMAs produce factors that can affect immune cells in their environment. Multiple studies have revealed that B-cell lymphopoiesis is inhibited in the presence of an adipocyte-rich environment [73,74]. One way adipocytes achieve this is by the recruitment of marrow-derived suppressor cells (MDSCs) [74,75]. MDSCs can also suppress T-cells and blunt their anti-tumor response by producing arginase-I [76,77]. Thus, studies have shown that adipose tissue is a major suppressor of B- and T-cell activity in the presence of cancer [78]. Another cell line that is affected by an adipocyte-rich environment is macrophages. While the direct effect of adipocyte secretions may promote M1 polarization and a pro-inflammatory type of macrophage in the absence of cancer [79,80], in the presence of cancer, adipocyte-rich environments seem to promote M2 polarization and the generation of tumor-associated macrophages (TAMs), which are anti-inflammatory and pro-cancer in nature [81,82,83].

### 3.4. Bone-Remodeling Signaling

BMA-expressed RANKL and adipocyte cytokines reprogram bone remodeling toward osteoclastogenesis and osteoblast suppression. Most bone metastasis results in osteolytic features in the bone. This is usually a result of promoting osteoclastogenesis [84]. BMAs are involved in this effect. Unlike other adipocytes around the body, BMAs are capable of producing receptor activator for nuclear factor κB ligand (RANKL), which is a major promoter of bone resorption and osteoclast differentiation [85,86]. Studies have shown that BMAs are the major source of *Tnfsf11*, the gene that encodes for RANKL, in mesenchymal cells [85]. While osteolytic remodeling has been the most intensively studied in the context of tumor-BMA interactions, osteoblastic activity is also profoundly influenced. BMA-secreted factors have been shown to induce changes in osteoblasts, one of which is converting them to a more adipocyte-like phenotype thereby shifting the remodeling balance and contributing to pathological bone turnover [87]. Additionally, the secretion of TNF-α and adipokines like adiponectin inhibits osteoblast differentiation and function by reducing acetyl-histone 3 levels and Runx2 transcription [88,89,90,91]. Thus, BMAs play a role in metastasis-associated bone remodeling.

### 3.5. Extracellular-Vesicle–Mediated Cargo Transfer

**Figure 1:**
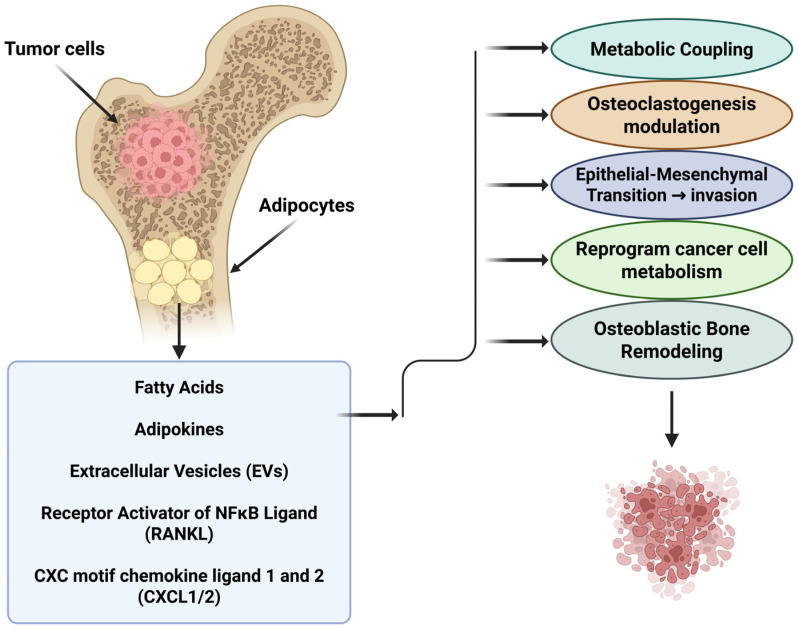
Mechanistic crosstalk between bone marrow adipocytes and tumor cells. Bone marrow Adipocytes supply fatty acids that fuel tumor oxidative phosphorylation and survival, while adipokines (e.g., leptin, IL-6, IL-1β) and extracellular vesicles (EVs) promote epithelial–mesenchymal transition (EMT), invasion, and metabolic rewiring. Adipocyte-derived RANKL and CXCL1/2 enhance osteoclastogenesis and inflammatory recruitment, accelerating bone resorption and liberating matrix growth factors that further support tumor outgrowth. In parallel, adipokines and EV cargo can bias osteoblast-lineage cells toward differentiation and matrix deposition, and couple osteoclast activity to compensatory bone formation, contributing to osteoblastic bone remodeling that often coexists with elevated osteoclast activity. Together, metabolic coupling, paracrine signaling, and bidirectional control of bone remodeling create a permissive niche that sustains metastatic expansion in bone. (EVs, extracellular vesicles; EMT, epithelial–mesenchymal transition; RANKL, receptor activator of NF-κB ligand.). Created in BioRender. Mohammad, K. (2025) https://BioRender.com/f8d3y6h.

The role of EVs in cellular communication, especially in relation to metastasis, has become the center of many studies [92,93]. Adipocytes are considered a major source for EVs [94], leading many studies to explore the role of adipocyte-derived EVs (ADEVs) in influencing metastasis [94,95,96,97,98,99]. Studies on melanoma reveal that ADEVs promote cancer cells’ migration properties and reprogram them to utilize fatty acid oxidation by transferring proteins and enzymes involved in the process to them [98]. In another study focusing on EVs secreted by pre-adipocytes, breast cancer cells had a dose-dependent enhancement to cell migration in-vitro due to the induction of the Wnt/β-catenin pathway, a signaling pathway associated with tumor growth [97]. Alternatively, EVs secreted by pre-adipocytes have been shown to negatively affect the migration capabilities as well as promote pro-apoptotic signaling molecules in ovarian cancer cells [100]. On the other hand, tumor-derived EVs have been shown to have profound effects on adipocytes, including mediating their transformation into cancer-associated adipocytes through EV miR-126 and miR-144, and promoting lipolysis and beige/brown differentiation of adipocytes by EV miR-155 [101,102]. These effects offer a glimpse into the remarkable depth of EVs as a medium for cellular communication (Figure 1).

## 4. Comparative Mechanisms Across Cancer Types and Tumor-Specific Features

In this section, we synthesize how BMAs influence bone metastasis across different cancers (cross-tumor synthesis) and also examine tumor-specific features that shape these interactions in unique ways. This dual approach helps identify both shared and distinct mechanisms by which BMAs contribute to the metastatic process. Across breast, prostate, lung cancers, and myeloma, BMAs engage tumors through shared conduits lipid transfer fueling FAO/OXPHOS (CD36/FABP4), adipokine/cytokine signaling (leptin, IL-6, IL-1β), immune modulation (MDSC/TAM polarization with impaired B/T cell function), RANKL-driven bone remodeling, and EV-mediated cargo exchange that converge on bone colonization, immune evasion, and therapy resistance. Notwithstanding these commonalities, cancer-specific features shape the net phenotype: breast and myeloma typically manifest osteolytic lesions; prostate appears osteoblastic yet retains osteoclast dependence; lung cancer exhibits an S100A8/A9–TLR4–IL-6 axis linking BMAs to angiogenesis and osteolysis. Direct cross-tumor comparisons remain limited by heterogeneous models and endpoints; few studies are designed for head-to-head evaluation across tumor types, a gap we highlight as a priority for future work.

### 4.1. Breast Cancer

The interaction between BMAs and breast cancer cells has been explored in multiple studies. A study on high-fat diet mice showed that elevated body fat composition stimulates the growth of osteolytic lesions and tibial destruction when breast cancer was inoculated into the tibia, compared to normal diet mice [103]. Another study specifically explored the effect of PPARγ, an enhancer of bone marrow adipogenesis [104], on breast cancer metastasis in high-fat diet mice [105]. In a murine breast cancer model, treatment with a PPARγ inhibitor significantly reduced osteolytic lesion formation. Although PPARγ signaling influences multiple pathways including aromatase activity, systemic inflammation, and adipogenesis these findings nonetheless support the concept that adipocyte differentiation and metabolic activity contribute to the formation of a pro-osteolytic microenvironment that facilitates metastatic outgrowth [105]. It is important to note that tumor colonization is a multi-step process distinct from initial dissemination, and BMA-mediated effects may primarily modulate the niche environment rather than the early steps of tumor cell spread.

### 4.2. Prostate Cancer

Multiple stromal components, including BMAs, have been implicated in prostate cancer’s tropism for the bone microenvironment, where they can regulate tumor cell homing, colonization, and growth. As reviewed by Salamanna et al. (2023), BMAs are emerging as critical mediators of bone metastasis across several tumor types, including prostate cancer. However, the subsequent discussion here focuses exclusively on prostate-specific mechanisms and data [14]. One study found that BMAs enhance prostate cancer spread by transporting fatty acids to tumor cells and upregulating IL-1β, heme oxygenase 1 (HMOX1), and FABP4 [25]. FTIR spectroscopy was also used to confirm the translocation of lipids from adipocytes to prostate cancer cells [106,107]. Another effect of BMAs is the release of chemokines like CXCL1 and CXCL2, which attract osteoclasts and promote bone remodeling [14]. Although prostate cancer is associated with osteoblastic lesions, it is understood that osteoclasts play an essential role in the generation of osteoblastic lesions [108,109]. Studies have also found that BMAs influence the metabolism in prostate cancer cells. The presence of adipocytes promotes hypoxia-inducible factor 1α (HIF-1α) activation in an oxygen-independent mechanism. This “pseudohypoxia” process influences pancreatic cancer cells to express the Warburg phenotype [110].

### 4.3. Lung Cancer

In the context of lung cancer, BMAs have been shown to contribute primarily to tumor progression and adaptation within the bone marrow niche, rather than initial homing. Adipocyte-derived cytokines (e.g., IL-6, CXCL12) and lipids support the survival, metabolic reprogramming, and chemoresistance of disseminated lung cancer cells once they have colonized the marrow. These effects are summarized in (Table 1). A 2020 study has revealed that a major factor that differentiates between bone-metastatic and non-bone metastatic small cell lung carcinoma cell lines is their ability to interact with BMAs via S100A8/A9 on cancer cells binding to toll-like receptor 4 (TLR4) on BMAs [111]. This binding upregulates the release of IL-6 from BMAs, a well-established growth factor for many tumor cells, as well as a driver of angiogenesis via VEGF expression [72,111,112]. Adipokines produced by BMAs have also demonstrated numerous significant effects in lung cancer studies. The previously mentioned leptin has been shown to promote tumor cell migration and survival by inducing EMT and inhibiting apoptosis, respectively [113,114]. Additionally, adiponectin promotes angiogenesis and osteolysis by enhancing VEGF expression and inducing osteoclastogenesis through the RANK/RANKL as well as the JAK/STAT pathways, respectively [115,116]. Lastly, resistin, another adipokine secreted by BMAs, promotes osteoclast activation, inhibits osteoblast activity, and enhances tumor microenvironment inflammation and angiogenesis by activating various signaling pathways like NF-κB, JAK/STAT, and PI3K/AKT [117,118,119,120].

### 4.4. Myeloma and Hematologic Malignancies

As previously mentioned, the interactions between multiple myeloma and BMAs have been explored, as multiple myeloma generates energy via oxidative phosphorylation, is enhanced by BMA-secreted leptin, and inhibits the secretion of adiponectin [49,56]. However, the effects of BMAs on hematologic malignancies have been shown to extend to chemotherapy resistance. The primary actors in this effect appear to be BMA-secreted leptin and adipsin. Adipsin is a serine protease secreted by adipocytes and associated with the alternative complement pathway, the secretion of insulin by the pancreatic β-cells, and the transport of glucose into adipocytes [56,121]. Both this factor and leptin have been implicated in modulating leukemic cell proliferation and survival within the bone marrow niche [122]. Another effect of BMAs that assists in chemotherapy resistance is their ability to sequester and metabolize daunorubicin, a chemotherapeutic agent used for acute myeloid and lymphoblastic leukemia [123]. Collectively, these findings support an important rather than crucial role for BMAs in shaping the bone marrow microenvironment in hematologic malignancies, including their potential contribution to chemoresistance.

While the effects of BMAs on tumor dissemination and colonization have been a major focus of research, there remains a relative lack of comparative studies that examine these mechanisms across tumor types under standardized experimental conditions. It is well established that many tumor cells disseminate early and enter dormancy in the bone marrow, but understanding how BMA-derived signals influence dormancy maintenance, reactivation, and outgrowth remains an important and under-explored question.

Table 1 summarizes the tumor-specific effects of BMAs on cancer cells.

**Table 1:** Tumor-specific effects of BMAs on cancer cells.

Cancer Type	Effect of BMAs on Tumor Cells	Mechanism	References
Breast cancer	Supports osteolytic lesion formation	Mice treated with PPARγ showed a marked decrease in osteolytic lesions	[105]
Prostate cancer	Enhance spread	Transport fatty acids to tumor cells and upregulates IL-1β, HMOX1, and FABP4	[25]
	Promote bone remodeling	Release chemokines that attract osteoclasts	[14]
	Influence metabolism	Promote HIF-1α activation	[110]
Lung cancer	Assist in tumor cell growth and angiogenesis	S100A8/9 or TLR4 interactions upregulate IL-6 release from BMAs	[72,111,112]
	Promote tumor cell migration and survival	Leptin-mediated induction of EMT and inhibition of apoptosis	[113,114]
	Promote angiogenesis and osteolysis	Adiponectin-mediated enhancement of VEGF expression and promotion of the RANK/RANKL and JAK/STAT pathways	[115,116]
	Promote osteoclast activation, inhibit osteoblast activity, enhance microenvironment inflammation, and promote angiogenesis	Resistin-mediated activation of NF-κB, JAK/STAT, and PI3K/AKT pathways	[117,118,119,120]
Myeloma and hematologic malignancies	Chemotherapy resistance	Leptin and adipsin-mediated induction of autophagy	[122]
		Sequestrating and metabolizing daunorubicin within BMAs	[123]

## 5. BMAs as Drivers of Therapeutic Resistance

In addition to what was mentioned previously, BMAs can impart chemotherapy resistance to cancer cells in other ways. BMAs’ ability to tilt cancer cells’ energy metabolism towards oxidative phosphorylation has been hypothesized to drive therapeutic resistance against oxidative stress-based chemotherapy [50]. On the other hand, studies have shown that the inhibition of fatty acid oxidation increases ROS production within leukemia cells and induces apoptosis of leukemia stem cells [124]. It is hypothesized that this occurs due to the leukemia cells’ reliance on fatty acid oxidation for the production of NADH and FADH_2_, as although BMAs influence these cells to use oxidative phosphorylation for energy production the main source of acetyl-CoA comes from fatty acid oxidation, while the pyruvate produced by glycolysis is mostly shunted towards lactate production [50]. Additionally, fatty acid oxidation within leukemia cells downregulates the proapoptotic Bak protein [125], while the activation of PPARγ upregulates the antiapoptotic BCL-2 [126]. It is important to note that in leukemia cells, BCL-2 also exhibits an antioxidant function by facilitating glutathione’s transport into the mitochondrial matrix as well as inhibiting reactive oxygen species (ROS) generation [50,127,128]. In addition to metabolic alterations, BMAs can induce the differentiation of cancer-associated fibroblasts (CAFs) [13]. Studies reveal that some adipocytes may dedifferentiate and turn into CAFs in the presence of cancer [129]. CAFs have been investigated as a major driver of chemotherapy resistance by producing different factors like IL-6, IL-17A, PDGF, and IGF, which activate many pathways that promote survival within cancer cells, like NF-κB and ERK1/2 pathways [130]. Adipokines secreted by BMAs can also assist in chemotherapy resistance. The previously mentioned resistin has been shown to be an inducer of ATP-binding cassette (ABC) transporter expression via promoting the demethylation of *ABCC5* and *ABCG2* genes as well as downregulating DNA methyltransferase 1 and 3a [131]. It is also capable of promoting the expression of antiapoptotic proteins like BCL-2 and BCL-xL as well as inhibiting proapoptotic proteins like Bak and Bax via the activation of the PI3K/Akt, ERK1/2, and NF-κB signaling pathways [131]. Adipocyte secretions have also been shown to elevate the expression of major vault proteins (MVPs) within cancer cells, like breast cancer [132]. These proteins allow cancer cells to transport chemotherapy agents like doxorubicin from the nucleus, which is their target site, into the cytoplasm, where they are then packaged into vesicles and excreted as EVs [133]. One study showed that MVP expression in obese patients was more prevalent than in non-obese patients [132]. All these effects of adipocytes, and BMAs in particular, can help explain the role of adipocytes in imparting chemotherapy resistance to cancer cells, and reveal the need for studies that stratify patients based on their adiposity, especially in the bone marrow, to guide therapy intensity.

Table 2 summarizes these mechanisms, detailing the molecules involved, tumor types in which they have been studied, and the experimental or clinical evidence supporting their roles. Each pathway presents therapeutic opportunities ranging from metabolic inhibition and adipokine blockade to RANKL targeting and modulation of extracellular vesicle exchange. By mapping these interactions to potential interventions, the table highlights both established and emerging avenues for disrupting the tumor-supportive functions of BMAs.

## 6. Experimental Models

### 6.1. In-Vitro Models

In vitro models provide controlled environments to study direct and indirect interactions between BMAs and tumor cells, focusing on cellular and molecular mechanisms:

#### 6.1.1. D Co-Cultures

These systems involve co-culturing BMAs, typically differentiated from MSCs or pre-adipocyte cell lines (e.g., 3T3-L1 or OP9), with tumor cell lines such as MDA-MB-231 (breast cancer), PC-3 (prostate cancer), or U266 (multiple myeloma) [147]. Direct co-cultures enable cell-cell contact, whereas transwell systems isolate paracrine effects, facilitating analysis of BMA-derived adipokines (e.g., leptin, adiponectin) and cytokines (e.g., IL-1β, CCL2) on tumor cell proliferation, migration, and invasion. These models are cost-effective but lack the complexity of the bone marrow niche [148].

#### 6.1.2. D Adipocyte–Bone Organoids

These advanced models incorporate BMAs, tumor cells, osteoblasts, osteoclasts, and extracellular matrix components (e.g., collagen, hydroxyapatite) to recapitulate the bone marrow microenvironment. Organoids are generated using scaffolds or hydrogel-based systems to mimic the spatial organization of bone tissue [149]. They enable study of BMA-tumor interactions in a physiologically relevant context, including effects on tumor cell adhesion, survival, and drug resistance. These models are beneficial for evaluating how BMAs contribute to the metastatic niche [147].

#### 6.1.3. Microfluidic Bone-on-a-Chip

Microfluidic platforms simulate the bone marrow microenvironment by integrating BMAs, tumor cells, and bone cells within microchannels that mimic vascular and interstitial flow. These systems allow real-time observation of cell migration, invasion, and lipid transfer under dynamic conditions. Bone-on-a-chip models offer high-throughput screening of BMA-derived factors and therapeutic responses, bridging the gap between 2D cultures and in vivo systems by replicating physiological fluid dynamics and cellular interactions, though they are limited by technical complexity and scalability [150].

### 6.2. In-Vivo Models

In vivo models capture the systemic and microenvironmental complexity of BMA-tumor interactions in bone metastases, often using murine systems to study tumor progression and bone remodeling. Ethical considerations, such as animal welfare during invasive procedures like irradiation or ovariectomy, must be addressed to ensure compliance with regulatory standards.

#### 6.2.1. High Marrow Fat Murine Models

These models increase bone marrow adiposity to study its impact on metastasis. Common approaches include:

##### Irradiation

Localized irradiation of bones (e.g., tibiae) induces marrow fat accumulation by damaging hematopoietic cells, creating an adipocyte-rich niche. Tumor cells are then injected (e.g., intracardiac or intratibial) to assess metastasis in this altered environment. However, irradiation may introduce confounding effects, such as inflammation or DNA damage, which could independently influence tumor behavior beyond BMA effects [88].

##### Ovariectomy

Surgical removal of ovaries in female mice mimics postmenopausal bone marrow adiposity and bone loss, increasing BMA content. This model is relevant for studying breast cancer metastasis in estrogen-deficient conditions [151].

##### PPARγ Agonists (e.g., Pioglitazone)

Pharmacological induction of adipogenesis using PPARγ agonists like pioglitazone increases BMA formation. These models evaluate how enhanced marrow adiposity promotes tumor colonization and osteolytic lesions, particularly in breast and prostate cancer [152].

#### 6.2.2. Patient-Derived Xenografts into Adipocyte-Rich Tibiae

Patient-derived xenografts (PDXs) involve implanting patient-derived tumor tissue into the tibiae of immunodeficient mice (e.g., NOD/SCID or NSG), which naturally have high marrow fat content. This model preserves tumor heterogeneity and is used to study BMA-tumor interactions in a clinically relevant context. Its application is currently limited to breast cancer due to challenges in establishing PDXs for other cancers (e.g., prostate or myeloma), including low engraftment rates, representing a research gap [152,153].

Beyond PDXs, immunodeficient mice are used for human tumor cell xenografts (e.g., via intracardiac or intratibial injection) to study bone metastases. Syngeneic models (e.g., 4T1 cells in C57BL/6 mice) incorporate immune responses, allowing investigation of inflammatory cytokines and immune cell recruitment in the BMA-tumor niche [154].

#### 6.2.3. Analytical Techniques

Advanced techniques complement these models to quantify BMA-tumor interactions and lipid dynamics in bone metastases.

Micro-MRI proton density fat fraction (PDFF) non-invasively measures bone marrow fat content by quantifying the proportion of fat signal relative to total signal (fat and water). It is used in vivo to assess BMA accumulation in high marrow fat models and correlate adiposity with metastatic burden. Standardization across different MRI platforms remains a challenge to ensure reproducibility in clinical settings [155].

Chemical Exchange Saturation Transfer (CEST) MRI detects specific molecular signals, such as those from lipids or metabolites, in the bone marrow. It provides insights into BMA composition and metabolic activity, helping identify lipid profiles associated with tumor progression. This technique requires specialized expertise and equipment, limiting its accessibility [156].

The Stable Isotope Pulse-Chase technique involves labeling lipids with stable isotopes (e.g., ^13^C-glucose or ^13^C-fatty acids) to trace their transfer from BMAs to tumor cells. Pulse-chase experiments quantify lipid uptake by tumor cells, elucidating how BMAs fuel tumor growth through lipid metabolism. Mass spectrometry or nuclear magnetic resonance (NMR) is used to track labeled metabolites, though these methods are costly and technically complex [157].

RNA sequencing identifies differentially expressed genes in BMA-tumor interactions, lipidomics profiles BMA-derived lipids, and immunohistochemistry visualizes BMA distribution and tumor cell infiltration in bone tissue. Bioluminescence imaging tracks tumor progression in vivo, while ELISA quantifies adipokine and cytokine levels [149,154].

Each model has strengths and limitations. In vitro models (2D co-cultures, 3D organoids, bone-on-a-chip) offer mechanistic insights but oversimplify the bone marrow niche. In vivo models capture physiological complexity but face challenges like species differences (xenografts) or limited clinical relevance (PDXs restricted to breast cancer). Analytical techniques like PDFF, CEST, and stable isotope pulse-chase enhance quantitative rigor but require specialized equipment. Combining multiple models and integrating multi-omics data provides a comprehensive understanding of BMA roles in bone metastases, paving the way for targeted therapies [158].

## 7. Clinical Evidence to Date

BMAs are critical components of the bone marrow microenvironment, influencing tumor progression and bone destruction in cancers such as breast, prostate, and multiple myeloma. Clinical evidence, though emerging, highlights their role in creating a tumor-supportive niche, with data drawn from imaging, biomarker studies, case reports, and patient cohorts [149,159].

### 7.1. Key Clinical Observations

Case reports, particularly in triple-negative breast cancer, describe marrow-dominant metastases characterized by extensive BMA replacement. Bone marrow biopsies reveal significant adipocyte infiltration alongside tumor cells, with MRI showing near-complete replacement of hematopoietic marrow by adipocytes. These findings suggest BMAs provide a lipid-rich niche supporting aggressive tumor growth, with elevated fatty acid-binding protein 4 (FABP4) expression in metastatic lesions [147,154].

Observational studies of patients with monoclonal gammopathy of undetermined significance (MGUS) demonstrate that those with high BMA density, assessed via MRI PDFF, progress more rapidly to multiple myeloma. Increased marrow adiposity correlates with elevated adipocyte-derived cytokines (e.g., IL-6, TNF-α) in bone marrow aspirates, promoting plasma cell proliferation and osteoclast activation [147,158].

Epidemiological data link higher marrow adiposity, which increases with age and obesity, to elevated risks of bone metastases. In breast cancer, postmenopausal women with high marrow fat fraction (via PDFF) show advanced disease stages and poorer prognosis. In prostate cancer, obese patients with increased marrow adiposity exhibit higher rates of bone metastases, potentially due to BMA-derived lipids and adipokines like leptin [154,160].

Elevated serum levels of BMA-derived adipokines (e.g., leptin, adiponectin) and cytokines (e.g., IL-1β, CCL2) are detected in patients with bone metastases, though these may not fully reflect bone marrow-specific dynamics. In multiple myeloma, higher leptin levels correlate with osteolytic lesions and disease activity, suggesting BMAs contribute to a pro-tumorigenic niche [147,154].

A 2024 study published in *Tomography* validated the reproducibility of MRI PDFF as an imaging biomarker in metastatic prostate cancer. Conducted across multiple centers, the study confirmed that PDFF measurements in the spine and pelvis reliably quantify marrow fat content, with high inter-observer agreement. Higher PDFF values were associated with increased osteolytic activity and tumor burden [156]. Preliminary radiomics studies have used PDFF thresholds to differentiate malignant from normal bone marrow. Machine learning algorithms analyzing PDFF patterns in MRI scans (e.g., fat fraction > 60%) show promise in identifying malignancy in breast and prostate cancers, enhancing diagnostic accuracy for bone metastases [161]. In breast cancer, patients with higher marrow adiposity exhibit reduced response to chemotherapy and targeted therapies (e.g., trastuzumab for HER2-positive disease), potentially due to BMA-mediated drug sequestration or metabolic support for tumor cells [154].

### 7.2. Limitations and Gaps

Many studies, including case reports and MGUS cohorts, involve limited patient numbers, reducing statistical power and generalizability [162]. Data is predominantly focused on breast cancer and multiple myeloma, with less evidence for prostate cancer and minimal data for other cancers (e.g., lung, renal cell carcinoma) that metastasize to bone [159]. Much of the evidence relies on correlative data from imaging (e.g., PDFF, CEST) or serum biomarkers, as bone marrow biopsies are invasive and less feasible for large-scale studies [163]. Few studies track BMA dynamics over time or in response to treatment, hindering insights into their role in disease progression or therapeutic response. Developing non-invasive methods for longitudinal BMA studies is needed to address this gap [164]. No clinical trials have specifically targeted BMAs or their secreted factors (e.g., leptin, FABP4) in bone metastases, limiting translation of preclinical findings [14].

Larger, prospective studies are needed to validate BMA contributions across diverse cancer types, particularly in underrepresented cancers like lung or renal cell carcinoma. Longitudinal studies tracking BMA density and biomarker profiles in MGUS-to-myeloma progression could inform risk stratification. Integrating advanced imaging (e.g., PDFF, CEST) with radiomics and molecular profiling of bone marrow aspirates may enhance diagnostic and prognostic tools. Clinical trials targeting adipokine signaling (e.g., leptin receptor antagonists) or lipid metabolism (e.g., FABP4 inhibitors) could offer novel strategies to disrupt the metastatic niche [165,166].

## 8. Therapeutic Targeting Strategies

### 8.1. Metabolic Blockade of BMA

Adipose triglyceride lipase (ATGL), encoded by the *PNPLA2* gene, mediates prostate cancer growth in vitro and in vivo. *PNPLA2* itself exhibits genomic and transcriptomic traits of an oncogene, and its expression has been correlated with worse overall survival [167]. Accordingly, ATGL is required for prostate cancer cell proliferation, survival, colony formation, and tumorgenicity, making it a potential therapeutic target in advanced prostate cancer [168,169,170]. In metastatic prostate cancer growing in bone, marrow adipocytes drive a glycolytic shift; pharmacologic inhibition of adipocyte ATGL blunts adipocyte-driven metabolic reprogramming signals in tumor cells [110]. Blocking adipocyte lipolysis with the ATGL inhibitor Atglistatin was found to decrease prostate cancer cell growth and colony formation in a dose-dependent manner. Both decreased proliferation (decreased p-HH3 levels) and increased cell death through increasing cleaved PARP, a marker of apoptosis [167]. Atglistatin reverses the tumor-induced inflammatory/lipolytic program in bone-marrow adipocytes and restores docetaxel (chemotherapy medication) sensitivity in prostate cancer models of the bone niche [134]. In metastatic prostate cancer growing in bone, marrow adipocytes drive a glycolytic/Warburg shift; pharmacologic inhibition of adipocyte ATGL (Atglistatin) blunts adipocyte-driven metabolic reprogramming signals in tumor cells [135]. Although a previous report in lung cancer cells observed the opposite effect, suggesting the potential for off-target effects [171]. However, increased colony formation when cells were switched to supraphysiological levels of glucose (25 mM) after ATGL knockdown may help explain the discrepancies in the field [167,171].

In several preclinical settings, Fatty Acid Synthase (FASN) inhibition has been shown to potentiate the effects of standard agents. Combinations with taxanes or platinum agents (chemotherapy drugs) produced greater tumor suppression than either agent alone [136]. FASN inhibitors block de-novo palmitate synthesis, which disrupts membrane lipid composition, oncogenic signaling, and can promote apoptosis or chemosensitization [137]. TVB-2640, a potent and reversible inhibitor of the FASN, was recently shown preclinically to synergize with other agents (for example, topoisomerase inhibitors) to reduce growth in aggressive models such as Triple-negative breast cancer brain metastasis models [138]. Toxicology, Pharmacokinetic and pharmacodynamic studies supported acceptable non-clinical safety for TVB-2640 and enabled first-in-human studies [139].

Despite promising preclinical data, there are no published trials combining BMA metabolic blockade with antiresorptives (bisphosphonates, denosumab), immunotherapy, or radiotherapy, highlighting a major translational gap. There is, however, promising data on combination metabolic–bone niche targeting that could synergize with standard of care but will require careful evaluation of safety given the systemic role of lipid metabolism.

### 8.2. PPARγ Modulation

PPARγ is a nuclear receptor that functions as a transcription factor. PPARγ agonists, such as thiazolidinediones (TZDs), have been shown to increase bone marrow adiposity. For instance, systemic PPARγ antagonism reduced metastatic tumor growth in adipocyte-rich bone in male rodents, indicating that PPARγ activation can promote marrow adiposity and potentially accelerate metastasis [105]. The study utilized bisphenol-A-diglycidyl ether (BADGE), a PPARγ antagonist, to assess its impact on tumor progression. Their findings indicate that high-fat diet (HFD)-induced bone marrow adiposity accelerates tumor progression and increases osteolytic lesions in metastatic models of breast cancer and melanoma [105]. On the other hand, Selective PPARγ modulators (SPPARMs) have been shown to induce a “browning” or multilocular phenotype in white adipose tissue (WAT), characterized by increased lipid oxidation [172]. The researchers administered rosiglitazone and pioglitazone, both PPARγ agonists, to adult mice and observed a significant transformation of unilocular adipocytes into multilocular ones. These multilocular adipocytes exhibited increased mitochondrial content and enhanced expression of uncoupling protein-1 (UCP-1, protein found in the inner mitochondrial membrane of brown and beige adipocytes, responsible for generating heat), PPARγ coactivator-1α (PGC-1α), and perilipin (crucial role in regulating lipid storage and metabolism within adipocytes). The study suggests that PPARγ activation induces a “browning” effect in white adipose tissue, characterized by increased mitochondrial activity and lipid [172]. Another study demonstrates that treatment with TZDs, such as rosiglitazone, induces deacetylation of PPARγ at specific lysine residues (K268 and K293). This modification promotes the browning of WAT, characterized by increased mitochondrial content and thermogenic gene expression [173]. The browning effect enhances energy expenditure and improves metabolic profiles in mice [173]. Cold-induced activation of brown adipose tissue (BAT) has been shown to inhibit tumor growth by triggering a metabolic shift in cancer cells [174]. Another study reported that thermogenesis in brown fat, triggered by cold exposure, can restrict tumor growth by depriving cancer cells of nutrients [175]. The study found that housing immunocompetent mice in a 4 °C environment, as opposed to 30 °C, led to an 80% reduction in tumor growth in a colorectal cancer model. This suggests that cold-induced BAT activation can suppress tumor growth [175]. The researchers observed that cold exposure reduced glucose uptake in tumor cells and impaired glycolysis, a process known as the Warburg effect. This metabolic shift indicates that BAT activation can influence tumor cell metabolism, making it less efficient [175]. While direct evidence for browning in BMAs is still limited, these findings in WAT suggest that similar metabolic changes could potentially decrease the tumor supportive capacity of BMAs. Targeting BMA PPARγ remains a potential strategy for modulating the bone marrow niche and influencing bone metastasis.

### 8.3. RANKL Pathway

RANKL is best known for regulating osteoclast differentiation and activity through its receptor RANK. Beyond bone remodeling, RANK is expressed by tumor cells in breast, prostate, and lung cancers, and RANKL has been shown to stimulate the migration of RANK-positive breast and prostate cancer cells, as well as melanoma cells in vitro [176,177,178,179]. These findings have led to the hypothesis that cancer cells expressing RANK may be drawn to bone, which contains high local concentrations of RANKL [176,177,178,179]. Although the RANKL pathway was originally targeted for osteoporosis and skeletal metastasis, recent studies implicating adipocyte-derived RANKL now place this pathway at the interface between bone marrow adiposity (BMA) and bone resorption [85,180]. Bone marrow adipocytes and adipogenic lineage precursors are now recognized as an important source of RANKL within the marrow niche [85,180]. Increased marrow adiposity, such as that seen in aging or estrogen deficiency, correlates with upregulated RANKL expression in marrow adipocytes [181], thereby amplifying osteoclastogenesis. These findings provide strong evidence that adipocyte-derived RANKL is functionally important in coupling fat accumulation to skeletal fragility [85,180,181]. Preclinical data supports this link. In a rabbit model of glucocorticoid-induced bone loss, prolonged methylprednisolone exposure increased marrow adiposity while reducing bone mineral density [144]. Early administration of zoledronic acid not only prevented further bone deterioration but also reversed the adipogenic shift, normalizing marrow fat fraction and adipocyte morphology to levels comparable to untreated controls [144]. Similarly, in a clinical study of postmenopausal women with osteoporosis, zoledronic acid reduced vertebral marrow fat content while enhancing bone mineral density and suppressing bone turnover [143]. In oncology trials, however, the RANKL inhibitor denosumab has shown more limited benefits [145,146]. In early-stage breast cancer, denosumab did not reduce disease recurrence [146], and in nonmetastatic castration-resistant prostate cancer, it produced only a modest improvement in bone metastasis–free survival [145]. Importantly, neither study directly assessed marrow adiposity as an endpoint. Taken together, these findings indicate that while the RANKL pathway was not originally identified through BMA research, adipocyte-derived RANKL provides a biological link between marrow fat and bone resorption. Anti-resorptives such as zoledronic acid and denosumab, although developed for osteoporosis and cancer, may exert secondary effects on marrow adiposity, a possibility that warrants further clinical investigation.

### 8.4. Adipokine/Chemokine Blockade

BMAs secrete key adipokines, including IL-6, leptin, and adiponectin, which have been linked to tumor progression mechanisms such as cell survival, angiogenesis, and migration [35]. BMA’s secretion of a variety of adipokines, cytokines, chemokines (including IL-6, CCL7, CXCL1/2), facilitates tumor cell recruitment, invasion, colonization, and proliferation in bone metastasis models (prostate, breast, melanoma) [14]. A comprehensive review highlights how BMAs can promote tumor metastasis through IL-6 signaling [12]. It notes that adipocyte-derived IL-6 enhances the invasion of breast and ovarian cancer cells, and that Tocilizumab, an IL-6R-blocking antibody, can reduce bone metastases and skeletal tumor growth in prostate cancer models. In prostate cancer cells (notably DU-145), Tocilizumab, when combined with the STAT-3 inhibitor Stattic, impaired proliferation, clonogenicity, invasion, and migration by targeting the IL-6/IL-6R/STAT-3 pathway [182]. In a mouse model of osteosarcoma, Tocilizumab alone reduced pulmonary metastases, and when used in combination with anlotinib, it significantly suppressed tumor growth and metastasis to the lungs, as well as decreased STAT3 activity [140]. Novel bispecific antibodies targeting both IL-6R and IL-8R showed superior inhibition of metastasis in mouse models compared to high-dose Tocilizumab combinations. These agents achieved a ~50% reduction in lung metastases at far lower doses, although they represent preclinical-stage innovations beyond plain Tocilizumab use [141]. BMAs secrete CXCL1 and CXCL2, which drive osteoclast maturation and contribute to tumor-induced bone degradation in metastatic prostate cancer models. Blocking these chemokines or the CXCR2 receptor significantly reduced adipocyte-induced osteoclast formation [142].

### 8.5. Lifestyle Modifications

**Figure 2:**
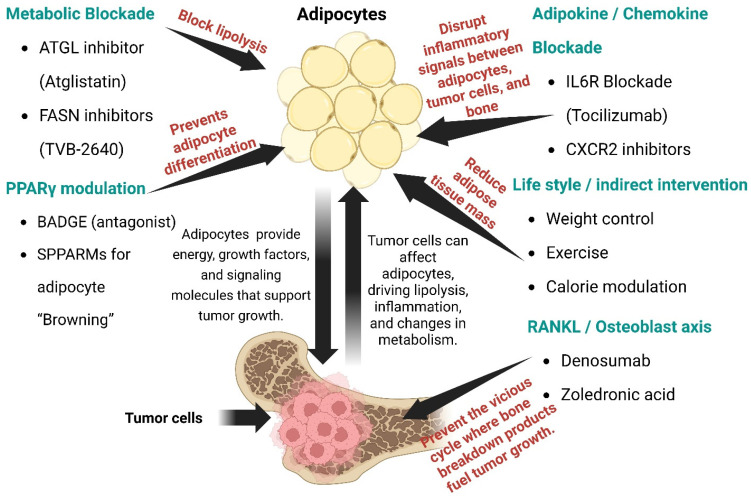
Therapeutic Strategies Targeting Bone Marrow Adipocyte–Tumor Interactions. Potential therapeutic approaches to disrupt adipocyte–tumor crosstalk include inhibition of adipocyte lipolysis (e.g., ATGL inhibitors), blockade of fatty acid synthesis (e.g., FASN inhibitors), modulation of PPARγ to prevent adipocyte differentiation or promote “browning,” and targeting the RANKL–osteoblast axis with antiresorptives (denosumab, zoledronic acid). Adipokine and chemokine signaling can be attenuated by IL-6R or CXCR2 inhibitors. Lifestyle interventions, including weight control, calorie modulation, and exercise, may indirectly reduce marrow adiposity. Together, these strategies aim to disrupt metabolic, endocrine, and inflammatory loops that sustain tumor progression in bone. Created in BioRender. Mohammad, K. (2025) https://BioRender.com/1qbfexp.

Excessive obesity leads to an increase in adiposity and BMA, which is thought to result from the preferential differentiation of mesenchymal stem cells into adipocytes rather than osteoblasts [183]. This shift contributes to bone fragility and osteoporosis [183]. In T2D, elevated circulating lipids, alterations in growth hormone, visceral adiposity, and hypoleptinemia have been identified as contributors to increased BMA, potentially mediating the skeletal fragility associated with diabetes [184]. Statins have shown promise in reducing BMA in osteonecrosis models, particularly in the context of steroid-induced osteonecrosis of the femoral head (SONFH) [185]. Research indicates that statins can decrease BMA and improve bone health by modulating lipid metabolism and inhibiting adipogenesis [185]. A study investigating the combined effects of statins and PPARγ inhibitors on steroid-induced osteonecrosis of the femoral head demonstrated that this combination therapy effectively reduced BMA and slowed SONFH progression in a rabbit model [185]. Caloric restriction (CR) has been associated with increased BMA and decreased bone mass in animal models [186]. One study reported that CR led to high marrow adiposity and low bone mass in growing mice, indicating that CR can promote adipogenesis within the bone marrow [186]. However, the impact of CR on bone health can vary depending on additional factors such as exercise [187]. For example, CR has been shown to reduce trabecular bone loss during aging and obesity, suggesting potential protective effects on bone health [187]. Exercise can also influence BMA, but its effects may differ under conditions of CR [186]. Specifically, a study examining exercise in the context of CR found that while exercise suppressed MAT, it did not prevent the overall increase in MAT associated with CR, highlighting the complex interactions between exercise, diet, and bone marrow adiposity [186] (Figure 2).

Despite emerging evidence that BMAs contribute to the establishment and progression of bone metastasis, therapeutic strategies targeting BMAs remain an area that is relatively underexplored in clinical trials. Most evidence derives from in vitro and animal studies, with limited clinical validation. Many studies that focus on breast, prostate, and ovarian cancer metastasizing to bone are available; however, there is a significant lack of studies that demonstrate the effects exclusive to BMAs and explore the therapeutic potential associated with them in combating bone metastasis in humans [15,25]. Thus, the need for further studies and research aimed at clarifying the mechanisms and therapeutic potential of targeting BMAs in bone metastasis is a field ripe for exploration.

## 9. Knowledge Gaps and Future Directions

### 9.1. Cellular Heterogeneity

Despite advances in BMA research, the cellular heterogeneity of BMAs remains poorly resolved. A study presents the first integrative high-resolution atlas of human bone and bone marrow, combining spatial transcriptomics and single-cell RNA-seq from femoral tissue [188]. It lays a foundational blueprint for exploring cellular architecture and molecular coordination in human bone and marrow and sets the stage for precise, spatially informed skeletal research [188]. Another study uncovers distinct MSC subtypes, including those with adipocytic signatures, and detailed micro-niches such as arterio-endosteal and adipocytic hematopoietic stem and progenitor cells niches [189]. Demonstrating how this atlas serves as a reference to explore niche composition, cell–cell neighborhoods, and pathological remodeling, particularly in AML [189]. An earlier, foundational study employed scRNA-seq and spatially resolved transcriptomics, particularly focusing on the human femoral head [190]. Discover key spatial niche features, such as an arterio-endosteal niche and an adipocytic niche for early hematopoietic Stem and Progenitor Cells [190]. Single-cell and spatial multi-omics technologies have yet to be systematically applied across skeletal compartments, leaving fundamental questions about BMA subtypes, developmental trajectories, and niche-specific roles unanswered. Generating comprehensive cellular maps will be essential to distinguish physiologically distinct adipocyte populations and their context-dependent functions.

### 9.2. Dynamic Lipidomics

Current knowledge of BMA lipid metabolism is largely derived from static snapshots. Real-time lipid tracing, particularly in patients, is critically lacking. Innovative metabolic flux approaches, such as stable isotope–based tracing combined with advanced lipidomics, could clarify how BMAs contribute to tumor lipid acquisition, systemic lipid pools, and energy balance. Stable-isotope metabolic tracing is a powerful in vivo tool for quantifying metabolic fluxes and dynamic turnover of nutrients in tumors, utilizing non-radioactive isotopes tracked by mass spectrometry or NMR [191]. Stable-isotope tracer methodology provides practical guidance for designing experiments and analyzing lipid metabolic fluxes in vivo, by tracing isotope incorporation into lipid molecules via NMR [192]. Translating these approaches into human studies is an urgent next step.

### 9.3. Immunometabolic Interface

The intersection of BMAs with immune regulation in the bone marrow microenvironment remains elusive. It is unclear whether BMAs support or impair immune surveillance, and how their metabolic products influence hematopoiesis, myeloid skewing, or antitumor immunity. Human BMAs exhibit a unique inflammatory profile that influences plasma cell function. This profile includes altered cytokine secretion and lipid metabolism, which can affect immune responses and hematopoietic processes. Human BMAs exhibit a unique inflammatory gene expression profile, characterized by increased levels of pro-inflammatory cytokines and ROS [193]. This inflammatory environment can influence the function and survival of plasma cells within the bone marrow niche, potentially impacting immune responses and hematopoiesis [193]. A review of immunometabolism examines how metabolic pathways influence immune cell function. It emphasizes the role of lipids in immune cell signaling and energy metabolism [194]. Applying these insights to BMAs can clarify how lipid metabolism in BMAs influences immune surveillance and hematopoietic stem cell niches [194]. Further studying these mechanisms could have major implications for understanding immunosenescence, bone marrow failure syndromes, and cancer progression.

### 9.4. Standardized Biomarkers

Currently, there is no consensus on non-invasive biomarkers for assessing BMA burden or function. PDFF cutoffs and circulating adipokines hold promise, but they require rigorous validation in diverse patient populations and across disease contexts. The ADIMOS Fracture Study found no significant association between bone marrow PDFF levels and incident fragility fractures in postmenopausal women, challenging PDFF’s utility in fracture risk prediction [195]. This offers a critical clinical insight into the translational limits of PDFF-based BMAT metrics for predicting fracture risk [195]. A 2022 review highlights MRI-PDFF as a versatile tool for fat quantification beyond the liver, including bone marrow, with applications in differentiating benign from malignant fractures and assessing age-related fatty replacement [196]. Providing a technical primer on PDFF usage across tissues and supports methodological expansion into marrow-specific applications [196]. Establishing standardized imaging and biochemical criteria would facilitate cross-study comparability and clinical translation.

### 9.5. Therapeutic Translation

Although preclinical studies suggest that metabolic inhibitors and bone-targeted agents can modulate BMA activity, translation into early-phase clinical trials has not yet occurred. AML demonstrates a critical dependence on fatty acid and lipid metabolism, particularly in the context of interactions with BMAs; targeting these metabolic dependencies may offer novel therapeutic opportunities [197]. Pharmacologic inhibition of fatty acid oxidation “FAO” (e.g., etomoxir) sensitizes human leukemia cells to apoptotic induction and decreases leukemic progenitor survival in vitro, supporting FAO inhibitors as a therapeutic class [198]. Denosumab is non-inferior to zoledronic acid for preventing skeletal events in myeloma, with better renal safety and standardized monthly dosing (120 mg) [199]. This establishes denosumab as a reliable backbone agent for future bone-targeted combinations in myeloma [199]. Developing rational combination strategies and incorporating biomarker endpoints will be key for advancing this field.

### 9.6. Pediatric and Sex Differences

Virtually unexplored, pediatric and sex-related differences in BMAs represent a profound but neglected dimension of research. The developmental stage, hormonal milieu, and pubertal transitions likely shape BMA abundance and function in ways distinct from those of adults. Similarly, sex-specific regulation by estrogens and androgens may underlie divergent skeletal and metabolic outcomes. A 2022 study mapped bone marrow adiposity using proton density fat fraction (PDFF) imaging across various anatomical sites, revealing distinct age- and sex-related patterns [200]. These findings highlight the need to consider age and sex as critical factors in BMAT research and clinical assessments [200]. A 2025 review discusses the broader context of skeletal dimorphism, suggesting that sex differences in BMAT may reflect distinct skeletal adaptation strategies [201]. Understanding these differences is crucial for developing sex-specific strategies in bone health management [201]. Addressing these gaps could reveal fundamental insights into biology and inform personalized approaches to bone health.

## 10. Conclusions

Bone marrow adipocytes (BMAs), once considered passive fillers, are now recognized as active and dynamic players in the metastatic cascade. Their ability to fuel tumor metabolism through fatty acid transfer, reshape the marrow niche via adipokines and cytokines, and modulate immune surveillance places them at the center of bone metastasis biology. Evidence across breast, prostate, lung cancers, and hematologic malignancies demonstrates that BMAs promote epithelial–mesenchymal transition, angiogenesis, osteoclastogenesis, and therapy resistance. Preclinical studies have highlighted promising interventions, including lipolysis inhibitors, PPARγ modulators, adipokine blockade, and RANKL-targeting agents; however, clinical translation remains limited. Emerging imaging and spatial multi-omics approaches reveal that BMA heterogeneity, lipid flux, and immunometabolic crosstalk are underexplored yet critically important. Addressing these gaps will require integrative models and well-designed clinical studies that stratify patients by marrow adiposity and metabolic state. Ultimately, targeting BMAs holds potential not only for disrupting metastatic progression but also for enhancing therapeutic response, offering a novel avenue to improve outcomes in cancers that preferentially colonize bone.

## Figures and Tables

**Table 2:** Mechanisms of bone marrow adipocyte–tumor crosstalk and therapeutic strategies.

Mechanism	Key Factors/Molecules	Tumor Types	Evidence	Potential Therapeutic Strategies
Metabolic Coupling	Free fatty acids, CD36, FABP4, PTHrP, lipolysis enzymes	Breast, prostate, multiple myeloma, leukemia	BMAs supply lipids to tumor cells → FAO/oxidative phosphorylation “OXPHOS” ↑, metabolic reprogramming [31,37,38,39,40,41,42,43,48,49,50]	ATGL inhibitors (Atglistatin), FASN inhibitors (TVB-2640), Fatty acid oxidation (FAO) inhibitors, metabolic flux monitoring [110,134,135,136,137,138,139]
Adipokine Signaling	Leptin, IL-6, TNF-α, adiponectin, IL-1β	Breast, prostate, lung, and myeloma	Promote EMT, survival, invasion, angiogenesis; suppress adiponectin’s anti-tumor effects [57,58,59,60,61,62,63,68,69,70]	IL-6R blockade (Tocilizumab), leptin receptor antagonists, anti-inflammatory strategies [140,141]
Immune Modulation	MDSCs, TAMs, arginase-I, cytokines (IL-6, IL-1β, TNF-α)	Breast, prostate, lung, melanoma	BMAs recruit suppressive immune cells → impaired B/T cell function [73,74,75,76,77,78,81,82,83]	CXCR2 inhibitors, immunotherapy combinations, targeting MDSC/TAM polarization [142]
Bone Remodeling	RANKL, osteoclast activation, osteolysis → growth factor release	Breast, prostate, myeloma	BMAs produce RANKL → osteoclastogenesis → bone resorption [85,86]	Denosumab (RANKL inhibitor), bisphosphonates (zoledronic acid), osteoclast-targeted therapy [143,144,145,146]
Extracellular Vesicles	ADEVs carrying lipids, enzymes, miRNAs (miR-155, miR-126, miR-144)	Breast, melanoma, ovarian, leukemia	EV-mediated metabolic reprogramming, cancer-associated adipocyte phenotype [94,95,96,97,98,99,101,102]	EV-targeted therapies, blocking vesicle uptake, lipid transfer inhibitors [17,18]
Therapy Resistance	Lipid-fueled oxidative metabolism, BCL-2/BCL-xL upregulation, drug sequestration	Leukemia, myeloma, breast cancer	BMAs confer chemotherapy resistance via autophagy, FAO, and drug sequestration [132,133]	Combination therapies (ATGL inhibitors + chemotherapy), ABC transporter inhibitors, metabolic targeting [131,132,133,134]

## References

[1] Fornetti J., Welm A.L., Stewart S.A. (2018). Understanding the Bone in Cancer Metastasis. J. Bone Miner. Res..

[2] Lee Y.-C., Gajdosik M.S., Josic D., Clifton J.G., Logothetis C., Yu-Lee L.-Y., Gallick G.E., Maity S.N., Lin S.-H. (2015). Secretome Analysis of an Osteogenic Prostate Tumor Identifies Complex Signaling Networks Mediating Cross-Talk of Cancer and Stromal Cells within the Tumor Microenvironment. Mol. Cell. Proteom..

[3] Lin S.-C., Lee Y.-C., Yu G., Cheng C.-J., Zhou X., Chu K., Murshed M., Le N.-T., Baseler L., Abe J.-I. (2017). Endothelial-to-Osteoblast Conversion Generates Osteoblastic Metastasis of Prostate Cancer. Dev. Cell.

[4] Nakashima K., Zhou X., Kunkel G., Zhang Z., Deng J.M., Behringer R.R., de Crombrugghe B. (2002). The Novel Zinc Finger-Containing Transcription Factor Osterix Is Required for Osteoblast Differentiation and Bone Formation. Cell.

[5] Lee Y.-C., Cheng C.-J., Bilen M.A., Lu J.-F., Satcher R.L., Yu-Lee L.-Y., Gallick G.E., Maity S.N., Lin S.-H. (2011). BMP4 Promotes Prostate Tumor Growth in Bone through Osteogenesis. Cancer Res..

[6] Delea T., Langer C., McKiernan J., Liss M., Edelsberg J., Brandman J., Sung J., Raut M., Oster G. (2004). The Cost of Treatment of Skeletal-Related Events in Patients with Bone Metastases from Lung Cancer. Oncology.

[7] Oster G., Lamerato L., Glass A.G., Richert-Boe K.E., Lopez A., Chung K., Richhariya A., Dodge T., Wolff G.G., Balakumaran A. (2013). Natural History of Skeletal-Related Events in Patients with Breast, Lung, or Prostate Cancer and Metastases to Bone: A 15-Year Study in Two Large US Health Systems. Support. Care Cancer.

[8] Giuliani N., Ferretti M., Bolzoni M., Storti P., Lazzaretti M., Dalla Palma B., Bonomini S., Martella E., Agnelli L., Neri A. (2012). Increased Osteocyte Death in Multiple Myeloma Patients: Role in Myeloma-Induced Osteoclast Formation. Leukemia.

[9] Cowan A.J., Green D.J., Kwok M., Lee S., Coffey D.G., Holmberg L.A., Tuazon S., Gopal A.K., Libby E.N. (2022). Diagnosis and Management of Multiple Myeloma: A Review. JAMA.

[10] Rajkumar S.V. (2024). Multiple Myeloma: 2024 Update on Diagnosis, Risk-Stratification, and Management. Am. J. Hematol..

[11] Wiedmeier-Nutor J.E., Fonseca R. (2025). Hyperdiploid Myeloma: The Silent Majority. Br. J. Haematol..

[12] Luo G., He Y., Yu X. (2018). Bone Marrow Adipocyte: An Intimate Partner With Tumor Cells in Bone Metastasis. Front. Endocrinol..

[13] Sato S., Hiruma T., Koizumi M., Yoshihara M., Nakamura Y., Tadokoro H., Motomatsu S., Yamanaka T., Washimi K., Okubo Y. (2023). Bone Marrow Adipocytes Induce Cancer-Associated Fibroblasts and Immune Evasion, Enhancing Invasion and Drug Resistance. Cancer Sci..

[14] Salamanna F., Contartese D., Errani C., Sartori M., Borsari V., Giavaresi G. (2023). Role of Bone Marrow Adipocytes in Bone Metastasis Development and Progression: A Systematic Review. Front. Endocrinol..

[15] Shin E., Koo J.S. (2020). The Role of Adipokines and Bone Marrow Adipocytes in Breast Cancer Bone Metastasis. Int. J. Mol. Sci..

[16] Zheng Y., Basel D., Chow S.-O., Fong-Yee C., Kim S., Buttgereit F., Dunstan C.R., Zhou H., Seibel M.J. (2014). Targeting IL-6 and RANKL Signaling Inhibits Prostate Cancer Growth in Bone. Clin. Exp. Metastasis.

[17] Moraes J.A., Encarnação C., Franco V.A., Xavier Botelho L.G., Rodrigues G.P., Ramos-Andrade I., Barja-Fidalgo C., Renovato-Martins M. (2021). Adipose Tissue-Derived Extracellular Vesicles and the Tumor Microenvironment: Revisiting the Hallmarks of Cancer. Cancers.

[18] Zhou C., Huang Y.-Q., Da M.-X., Jin W.-L., Zhou F.-H. (2023). Adipocyte-Derived Extracellular Vesicles: Bridging the Communications between Obesity and Tumor Microenvironment. Discov. Oncol..

[19] Cho J.A., Park H., Lim E.H., Lee K.W. (2012). Exosomes from Breast Cancer Cells Can Convert Adipose Tissue-Derived Mesenchymal Stem Cells into Myofibroblast-like Cells. Int. J. Oncol..

[20] Tratwal J., Rojas-Sutterlin S., Bataclan C., Blum S., Naveiras O. (2021). Bone Marrow Adiposity and the Hematopoietic Niche: A Historical Perspective of Reciprocity, Heterogeneity, and Lineage Commitment. Best Pract. Res. Clin. Endocrinol. Metab..

[21] Trotter T.N., Gibson J.T., Sherpa T.L., Gowda P.S., Peker D., Yang Y. (2016). Adipocyte-Lineage Cells Support Growth and Dissemination of Multiple Myeloma in Bone. Am. J. Pathol..

[22] Shafat M.S., Oellerich T., Mohr S., Robinson S.D., Edwards D.R., Marlein C.R., Piddock R.E., Fenech M., Zaitseva L., Abdul-Aziz A. (2017). Leukemic Blasts Program Bone Marrow Adipocytes to Generate a Protumoral Microenvironment. Blood.

[23] Jafari A., Fairfield H., Andersen T.L., Reagan M.R. (2021). Myeloma-Bone Marrow Adipocyte Axis in Tumour Survival and Treatment Response. Br. J. Cancer.

[24] Scheller E.L., Doucette C.R., Learman B.S., Cawthorn W.P., Khandaker S., Schell B., Wu B., Ding S.-Y., Bredella M.A., Fazeli P.K. (2015). Region-Specific Variation in the Properties of Skeletal Adipocytes Reveals Regulated and Constitutive Marrow Adipose Tissues. Nat. Commun..

[25] Herroon M.K., Rajagurubandara E., Hardaway A.L., Powell K., Turchick A., Feldmann D., Podgorski I. (2013). Bone Marrow Adipocytes Promote Tumor Growth in Bone via FABP4-Dependent Mechanisms. Oncotarget.

[26] Craft C.S., Li Z., MacDougald O.A., Scheller E.L. (2018). Molecular Differences between Subtypes of Bone Marrow Adipocytes. Curr. Mol. Biol. Rep..

[27] Singhal V., Torre Flores L.P., Stanford F.C., Toth A.T., Carmine B., Misra M., Bredella M.A. (2018). Differential Associations between Appendicular and Axial Marrow Adipose Tissue with Bone Microarchitecture in Adolescents and Young Adults with Obesity. Bone.

[28] Vandereyken K., Sifrim A., Thienpont B., Voet T. (2023). Methods and Applications for Single-Cell and Spatial Multi-Omics. Nat. Rev. Genet..

[29] Cabia B., Andrade S., Carreira M.C., Casanueva F.F., Crujeiras A.B. (2016). A Role for Novel Adipose Tissue-Secreted Factors in Obesity-Related Carcinogenesis. Obes. Rev..

[30] Song Y., Na H., Lee S.E., Kim Y.M., Moon J., Nam T.W., Ji Y., Jin Y., Park J.H., Cho S.C. (2024). Dysfunctional Adipocytes Promote Tumor Progression through YAP/TAZ-Dependent Cancer-Associated Adipocyte Transformation. Nat. Commun..

[31] Panaroni C., Fulzele K., Mori T., Siu K.T., Onyewadume C., Maebius A., Raje N. (2022). Multiple Myeloma Cells Induce Lipolysis in Adipocytes and Uptake Fatty Acids through Fatty Acid Transporter Proteins. Blood.

[32] Wu X., Li F., Dang L., Liang C., Lu A., Zhang G. (2020). RANKL/RANK System-Based Mechanism for Breast Cancer Bone Metastasis and Related Therapeutic Strategies. Front. Cell Dev. Biol..

[33] Fajol A., Komaba H. (2019). Additional Evidence for the Role of Parathyroid Hormone in Adipose Tissue Browning. EBioMedicine.

[34] Mukherjee S., Aseer K.R., Yun J.W. (2020). Roles of Macrophage Colony Stimulating Factor in White and Brown Adipocytes. Biotechnol. Bioprocess Eng..

[35] Liu C., Zhao Q., Yu X. (2020). Bone Marrow Adipocytes, Adipocytokines, and Breast Cancer Cells: Novel Implications in Bone Metastasis of Breast Cancer. Front. Oncol..

[36] Soni S., Torvund M., Mandal C.C. (2021). Molecular Insights into the Interplay between Adiposity, Breast Cancer and Bone Metastasis. Clin. Exp. Metastasis.

[37] Martinez-Outschoorn U.E., Pestell R.G., Howell A., Tykocinski M.L., Nagajyothi F., Machado F.S., Tanowitz H.B., Sotgia F., Lisanti M.P. (2011). Energy Transfer in “Parasitic” Cancer Metabolism: Mitochondria Are the Powerhouse and Achilles’ Heel of Tumor Cells. Cell Cycle.

[38] Wu Q., Li B., Li Z., Li J., Sun S., Sun S. (2019). Cancer-Associated Adipocytes: Key Players in Breast Cancer Progression. J. Hematol. Oncol..

[39] Wang T., Zhou D., Hong Z. (2025). Sarcopenia and Cachexia: Molecular Mechanisms and Therapeutic Interventions. MedComm (2020).

[40] Li Y., Li Z., Ngandiri D.A., Llerins Perez M., Wolf A., Wang Y. (2022). The Molecular Brakes of Adipose Tissue Lipolysis. Front. Physiol..

[41] Sun S., Wang Z., Yao F., Sun K., Li Z., Sun S., Li C. (2023). Breast Cancer Cell-Derived Exosome-Delivered microRNA-155 Targets UBQLN1 in Adipocytes and Facilitates Cancer Cachexia-Related Fat Loss. Hum. Mol. Genet..

[42] Librizzi M., Naselli F., Abruscato G., Luparello C., Caradonna F. (2023). Parathyroid Hormone Related Protein (PTHrP)-Associated Molecular Signatures in Tissue Differentiation and Non-Tumoral Diseases. Biology.

[43] Mukherjee A., Bilecz A.J., Lengyel E. (2022). The Adipocyte Microenvironment and Cancer. Cancer Metastasis Rev..

[44] Gyamfi J., Yeo J.H., Kwon D., Min B.S., Cha Y.J., Koo J.S., Jeong J., Lee J., Choi J. (2021). Interaction between CD36 and FABP4 Modulates Adipocyte-Induced Fatty Acid Import and Metabolism in Breast Cancer. NPJ Breast Cancer.

[45] Lemberger L., Wagner R., Heller G., Pils D., Grunt T.W. (2022). Pharmacological Inhibition of Lipid Import and Transport Proteins in Ovarian Cancer. Cancers.

[46] Zhao G., Cardenas H., Matei D. (2019). Ovarian Cancer-Why Lipids Matter. Cancers.

[47] Yu C., Niu X., Du Y., Chen Y., Liu X., Xu L., Iwakura Y., Ma X., Li Y., Yao Z. (2020). IL-17A Promotes Fatty Acid Uptake through the IL-17A/IL-17RA/p-STAT3/FABP4 Axis to Fuel Ovarian Cancer Growth in an Adipocyte-Rich Microenvironment. Cancer Immunol. Immunother..

[48] Wang Y., Patti G.J. (2023). The Warburg Effect: A Signature of Mitochondrial Overload. Trends Cell Biol..

[49] Fairfield H., Karam M., Schimelman A., Qiang Y.-W., Reagan M.R. (2024). Adipocytes and Metabolism: Contributions to Multiple Myeloma. J. Bone Oncol..

[50] Tabe Y., Konopleva M., Andreeff M. (2020). Fatty Acid Metabolism, Bone Marrow Adipocytes, and AML. Front. Oncol..

[51] Deng T., Lyon C.J., Bergin S., Caligiuri M.A., Hsueh W.A. (2016). Obesity, Inflammation, and Cancer. Annu. Rev. Pathol..

[52] Zhou B.O., Yu H., Yue R., Zhao Z., Rios J.J., Naveiras O., Morrison S.J. (2017). Bone Marrow Adipocytes Promote the Regeneration of Stem Cells and Haematopoiesis by Secreting SCF. Nat. Cell Biol..

[53] Maroni P. (2020). Leptin, Adiponectin, and Sam68 in Bone Metastasis from Breast Cancer. Int. J. Mol. Sci..

[54] Gu L., Wang C.-D., Cao C., Cai L.-R., Li D.-H., Zheng Y.-Z. (2019). Association of Serum Leptin with Breast Cancer: A Meta-Analysis. Medicine.

[55] Cha Y.J., Koo J.S. (2019). Roles of Omental and Bone Marrow Adipocytes in Tumor Biology. Adipocyte.

[56] Morris E.V., Edwards C.M. (2018). Adipokines, Adiposity, and Bone Marrow Adipocytes: Dangerous Accomplices in Multiple Myeloma. J. Cell. Physiol..

[57] Gorrab A., Pagano A., Ayed K., Chebil M., Derouiche A., Kovacic H., Gati A. (2021). Leptin Promotes Prostate Cancer Proliferation and Migration by Stimulating STAT3 Pathway. Nutr. Cancer.

[58] Bowers L.W., Rossi E.L., McDonell S.B., Doerstling S.S., Khatib S.A., Lineberger C.G., Albright J.E., Tang X., deGraffenried L.A., Hursting S.D. (2018). Leptin Signaling Mediates Obesity-Associated CSC Enrichment and EMT in Preclinical TNBC Models. Mol. Cancer Res..

[59] Juárez-Cruz J.C., Zuñiga-Eulogio M.D., Olea-Flores M., Castañeda-Saucedo E., Mendoza-Catalán M.Á., Ortuño-Pineda C., Moreno-Godínez M.E., Villegas-Comonfort S., Padilla-Benavides T., Navarro-Tito N. (2019). Leptin Induces Cell Migration and Invasion in a FAK-Src-Dependent Manner in Breast Cancer Cells. Endocr. Connect..

[60] Bader J.E., Wolf M.M., Lupica-Tondo G.L., Madden M.Z., Reinfeld B.I., Arner E.N., Hathaway E.S., Steiner K.K., Needle G.A., Hatem Z. (2024). Obesity Induces PD-1 on Macrophages to Suppress Anti-Tumour Immunity. Nature.

[61] Tsai C.-F., Chen J.-H., Wu C.-T., Chang P.-C., Wang S.-L., Yeh W.-L. (2019). Induction of Osteoclast-like Cell Formation by Leptin-Induced Soluble Intercellular Adhesion Molecule Secreted from Cancer Cells. Ther. Adv. Med. Oncol..

[62] He J.-Y., Wei X.-H., Li S.-J., Liu Y., Hu H.-L., Li Z.-Z., Kuang X.-H., Wang L., Shi X., Yuan S.-T. (2018). Adipocyte-Derived IL-6 and Leptin Promote Breast Cancer Metastasis via Upregulation of Lysyl Hydroxylase-2 Expression. Cell Commun. Signal..

[63] Du H., Pang M., Hou X., Yuan S., Sun L. (2017). PLOD2 in Cancer Research. Biomed. Pharmacother..

[64] Habanjar O., Bingula R., Decombat C., Diab-Assaf M., Caldefie-Chezet F., Delort L. (2023). Crosstalk of Inflammatory Cytokines within the Breast Tumor Microenvironment. Int. J. Mol. Sci..

[65] Kong G., Jiang Y., Sun X., Cao Z., Zhang G., Zhao Z., Zhao Y., Yu Q., Cheng G. (2017). Irisin Reverses the IL-6 Induced Epithelial-Mesenchymal Transition in Osteosarcoma Cell Migration and Invasion through the STAT3/Snail Signaling Pathway. Oncol. Rep..

[66] Saito K., Mitsui A., Sumardika I.W., Yokoyama Y., Sakaguchi M., Kondo E. (2021). PLOD2-Driven IL-6/STAT3 Signaling Promotes the Invasion and Metastasis of Oral Squamous Cell Carcinoma via Activation of Integrin Β1. Int. J. Oncol..

[67] Viveiros M.M.H., de Melo Viveiros M.E., Silva M.G., Kaneno R., Avelino N.P., Rainho C.A., Schellini S.A. (2022). Expression of Inflammatory Cytokines in Mesenchymal Stem Cells Derived from Proximal Humerus Fractures. Stem Cell Investig..

[68] Morris E.V., Suchacki K.J., Hocking J., Cartwright R., Sowman A., Gamez B., Lea R., Drake M.T., Cawthorn W.P., Edwards C.M. (2020). Myeloma Cells Down-Regulate Adiponectin in Bone Marrow Adipocytes Via TNF-Alpha. J. Bone Miner. Res..

[69] Zhang Z., Du J., Shi H., Wang S., Yan Y., Xu Q., Zhou S., Zhao Z., Mu Y., Qian C. (2022). Adiponectin Suppresses Tumor Growth of Nasopharyngeal Carcinoma through Activating AMPK Signaling Pathway. J. Transl. Med..

[70] Nigro E., Daniele A., Salzillo A., Ragone A., Naviglio S., Sapio L. (2021). AdipoRon and Other Adiponectin Receptor Agonists as Potential Candidates in Cancer Treatments. Int. J. Mol. Sci..

[71] Tulotta C., Ottewell P. (2018). The Role of IL-1B in Breast Cancer Bone Metastasis. Endocr. Relat. Cancer.

[72] Li J., Wu J., Xie Y., Yu X. (2024). Bone Marrow Adipocytes and Lung Cancer Bone Metastasis: Unraveling the Role of Adipokines in the Tumor Microenvironment. Front. Oncol..

[73] Bilwani F.A., Knight K.L. (2012). Adipocyte-Derived Soluble Factor(s) Inhibits Early Stages of B Lymphopoiesis. J. Immunol..

[74] Kennedy D.E., Knight K.L. (2015). Inhibition of B Lymphopoiesis by Adipocytes and IL-1-Producing Myeloid-Derived Suppressor Cells. J. Immunol..

[75] Perico M.E., Maluta T., Conti G., Vella A., Provezza L., Cestari T., De Cao G., Segalla L., Tecchio C., Benedetti F. (2021). The Cross-Talk between Myeloid and Mesenchymal Stem Cells of Human Bone Marrow Represents a Biomarker of Aging That Regulates Immune Response and Bone Reabsorption. Cells.

[76] Rodríguez P.C., Ochoa A.C. (2006). T Cell Dysfunction in Cancer: Role of Myeloid Cells and Tumor Cells Regulating Amino Acid Availability and Oxidative Stress. Semin. Cancer Biol..

[77] Wu Y., Yi M., Niu M., Mei Q., Wu K. (2022). Myeloid-Derived Suppressor Cells: An Emerging Target for Anticancer Immunotherapy. Mol. Cancer.

[78] Huang R., Wang Z., Hong J., Wu J., Huang O., He J., Chen W., Li Y., Chen X., Shen K. (2023). Targeting Cancer-Associated Adipocyte-Derived CXCL8 Inhibits Triple-Negative Breast Cancer Progression and Enhances the Efficacy of Anti-PD-1 Immunotherapy. Cell Death Dis..

[79] Engin A.B. (2024). Message Transmission Between Adipocyte and Macrophage in Obesity. Adv. Exp. Med. Biol..

[80] Piotrowska K., Zgutka K., Tkacz M., Tarnowski M. (2023). Physical Activity as a Modern Intervention in the Fight against Obesity-Related Inflammation in Type 2 Diabetes Mellitus and Gestational Diabetes. Antioxidants.

[81] Chung H.-Y., Kim J.-H., Han I.-H., Ryu J.-S. (2020). Polarization of M2 Macrophages by Interaction between Prostate Cancer Cells Treated with Trichomonas Vaginalis and Adipocytes. Korean J. Parasitol..

[82] Zhao C., Zeng N., Zhou X., Tan Y., Wang Y., Zhang J., Wu Y., Zhang Q. (2023). CAA-Derived IL-6 Induced M2 Macrophage Polarization by Activating STAT3. BMC Cancer.

[83] Zheng Q., Zhang J., Liu Y., Dong W., Dai X., Du X., Gu D. (2023). LINC01119 Encapsulated by Cancer-Associated Adipocytes-Derived Exosomes Promotes M2 Polarization of Macrophages to Induce Immune Escape in Ovarian Cancer in a 3D Co-Culture Cell-Based Model. Clin. Transl. Oncol..

[84] He Y., Luo W., Liu Y., Wang Y., Ma C., Wu Q., Tian P., He D., Jia Z., Lv X. (2022). IL-20RB Mediates Tumoral Response to Osteoclastic Niches and Promotes Bone Metastasis of Lung Cancer. J. Clin. Investig..

[85] Yu W., Zhong L., Yao L., Wei Y., Gui T., Li Z., Kim H., Holdreith N., Jiang X., Tong W. (2021). Bone Marrow Adipogenic Lineage Precursors Promote Osteoclastogenesis in Bone Remodeling and Pathologic Bone Loss. J. Clin. Investig..

[86] Fan Y., Hanai J.-I., Le P.T., Bi R., Maridas D., DeMambro V., Figueroa C.A., Kir S., Zhou X., Mannstadt M. (2017). Parathyroid Hormone Directs Bone Marrow Mesenchymal Cell Fate. Cell Metab..

[87] Clabaut A., Grare C., Rolland-Valognes G., Letarouilly J.-G., Bourrier C., Andersen T.L., Sikjær T., Rejnmark L., Ejersted C., Pastoureau P. (2021). Adipocyte-Induced Transdifferentiation of Osteoblasts and Its Potential Role in Age-Related Bone Loss. PLoS ONE.

[88] Liu H., Liu L., Rosen C.J. (2025). Bone Marrow Adipocytes as Novel Regulators of Metabolic Homeostasis: Clinical Consequences of Bone Marrow Adiposity. Curr. Obes. Rep..

[89] Deepika F., Bathina S., Armamento-Villareal R. (2023). Novel Adipokines and Their Role in Bone Metabolism: A Narrative Review. Biomedicines.

[90] Burkhardt L.-M., Bucher C.H., Löffler J., Rinne C., Duda G.N., Geissler S., Schulz T.J., Schmidt-Bleek K. (2023). The Benefits of Adipocyte Metabolism in Bone Health and Regeneration. Front. Cell Dev. Biol..

[91] Abuna R.P.F., Almeida L.O., Souza A.T.P., Fernandes R.R., Sverzut T.F.V., Rosa A.L., Beloti M.M. (2021). Osteoporosis and Osteoblasts Cocultured with Adipocytes Inhibit Osteoblast Differentiation by Downregulating Histone Acetylation. J. Cell. Physiol..

[92] Wang S.E. (2020). Extracellular Vesicles and Metastasis. Cold Spring Harb. Perspect. Med..

[93] Urabe F., Patil K., Ramm G.A., Ochiya T., Soekmadji C. (2021). Extracellular Vesicles in the Development of Organ-Specific Metastasis. J. Extracell. Vesicles.

[94] Thomou T., Mori M.A., Dreyfuss J.M., Konishi M., Sakaguchi M., Wolfrum C., Rao T.N., Winnay J.N., Garcia-Martin R., Grinspoon S.K. (2017). Adipose-Derived Circulating miRNAs Regulate Gene Expression in Other Tissues. Nature.

[95] Flaherty S.E., Grijalva A., Xu X., Ables E., Nomani A., Ferrante A. (2019). A Lipase-Independent Pathway of Lipid Release and Immune Modulation by Adipocytes. Science.

[96] Valenzuela Alvarez M., Gutierrez L.M., Correa A., Lazarowski A., Bolontrade M.F. (2019). Metastatic Niches and the Modulatory Contribution of Mesenchymal Stem Cells and Its Exosomes. Int. J. Mol. Sci..

[97] Lin R., Wang S., Zhao R.C. (2013). Exosomes from Human Adipose-Derived Mesenchymal Stem Cells Promote Migration through Wnt Signaling Pathway in a Breast Cancer Cell Model. Mol. Cell. Biochem..

[98] Clement E., Lazar I., Attané C., Carrié L., Dauvillier S., Ducoux-Petit M., Esteve D., Menneteau T., Moutahir M., Le Gonidec S. (2020). Adipocyte Extracellular Vesicles Carry Enzymes and Fatty Acids That Stimulate Mitochondrial Metabolism and Remodeling in Tumor Cells. EMBO J..

[99] Giordano C., La Camera G., Gelsomino L., Barone I., Bonofiglio D., Andò S., Catalano S. (2020). The Biology of Exosomes in Breast Cancer Progression: Dissemination, Immune Evasion and Metastatic Colonization. Cancers.

[100] Reza A.M.M.T., Choi Y.-J., Yasuda H., Kim J.-H. (2016). Human Adipose Mesenchymal Stem Cell-Derived Exosomal-miRNAs Are Critical Factors for Inducing Anti-Proliferation Signalling to A2780 and SKOV-3 Ovarian Cancer Cells. Sci. Rep..

[101] Yang E., Wang X., Gong Z., Yu M., Wu H., Zhang D. (2020). Exosome-Mediated Metabolic Reprogramming: The Emerging Role in Tumor Microenvironment Remodeling and Its Influence on Cancer Progression. Signal Transduct. Target. Ther..

[102] Liu Y., Wang M., Deng T., Liu R., Ning T., Bai M., Ying G., Zhang H., Ba Y. (2022). Exosomal miR-155 from Gastric Cancer Induces Cancer-Associated Cachexia by Suppressing Adipogenesis and Promoting Brown Adipose Differentiation via C/EPBβ. Cancer Biol. Med..

[103] Huang M., Liu H., Zhu L., Li X., Li J., Yang S., Liu D., Song X., Yokota H., Zhang P. (2021). Mechanical Loading Attenuates Breast Cancer-Associated Bone Metastasis in Obese Mice by Regulating the Bone Marrow Microenvironment. J. Cell. Physiol..

[104] Ren H., Mao K., Yuan X., Mu Y., Zhao S., Fan X., Zhu L., Ye Z., Lan J. (2024). AN698/40746067 Suppresses Bone Marrow Adiposity to Ameliorate Hyperlipidemia-Induced Osteoporosis through Targeted Inhibition of ENTR1. Biomed. Pharmacother..

[105] Gaculenko A., Gregoric G., Popp V., Seyler L., Ringer M., Kachler K., Wu Z., Kisel W., Hofbauer C., Hofbauer L.C. (2021). Systemic PPARγ Antagonism Reduces Metastatic Tumor Progression in Adipocyte-Rich Bone in Excess Weight Male Rodents. J. Bone Miner. Res..

[106] Mitura P., Paja W., Klebowski B., Wronecki L., Godzisz M., Sudoł D., Bar K., Depciuch J. (2025). FTIR Markers of Prostate Cancer Tissue and Their Correlation With Medical Parameters of Tumor Aggressiveness. J. Biophotonics.

[107] Roman M., Wrobel T.P., Panek A., Kwiatek W.M. (2024). High-Definition FT-IR Reveals a Synergistic Effect on Lipid Accumulation in Prostate Cancer Cells Induced by a Combination of X-Rays and Radiosensitizing Drugs. Biochim. Biophys. Acta Mol. Cell Biol. Lipids.

[108] Roato I., D’Amelio P., Gorassini E., Grimaldi A., Bonello L., Fiori C., Delsedime L., Tizzani A., De Libero A., Isaia G. (2008). Osteoclasts Are Active in Bone Forming Metastases of Prostate Cancer Patients. PLoS ONE.

[109] Yu L., Sui B., Fan W., Lei L., Zhou L., Yang L., Diao Y., Zhang Y., Li Z., Liu J. (2021). Exosomes Derived from Osteogenic Tumor Activate Osteoclast Differentiation and Concurrently Inhibit Osteogenesis by Transferring COL1A1-Targeting miRNA-92a-1-5p. J. Extracell. Vesicles.

[110] Diedrich J.D., Rajagurubandara E., Herroon M.K., Mahapatra G., Hüttemann M., Podgorski I. (2016). Bone Marrow Adipocytes Promote the Warburg Phenotype in Metastatic Prostate tumorsviaHIF-1α Activation. Oncotarget.

[111] Luo G., Tang M., Zhao Q., Lu L., Xie Y., Li Y., Liu C., Tian L., Chen X., Yu X. (2020). Bone Marrow Adipocytes Enhance Osteolytic Bone Destruction by Activating 1q21.3(S100A7/8/9-IL6R)-TLR4 Pathway in Lung Cancer. J. Cancer Res. Clin. Oncol..

[112] Yao X., Huang J., Zhong H., Shen N., Faggioni R., Fung M., Yao Y. (2014). Targeting Interleukin-6 in Inflammatory Autoimmune Diseases and Cancers. Pharmacol. Ther..

[113] Xu M., Cao F.-L., Li N., Gao X., Su X., Jiang X. (2018). Leptin Induces Epithelial-to-Mesenchymal Transition via Activation of the ERK Signaling Pathway in Lung Cancer Cells. Oncol. Lett..

[114] Philp L.K., Rockstroh A., Sadowski M.C., Taherian Fard A., Lehman M., Tevz G., Libério M.S., Bidgood C.L., Gunter J.H., McPherson S. (2021). Leptin Antagonism Inhibits Prostate Cancer Xenograft Growth and Progression. Endocr. Relat. Cancer.

[115] Maroni P., Luzzati A., Perrucchini G., Cannavò L., Bendinelli P. (2020). Leptin, Leptin Receptor, KHDRBS1 (KH RNA Binding Domain Containing, Signal Transduction Associated 1), and Adiponectin in Bone Metastasis from Breast Carcinoma: An Immunohistochemical Study. Biomedicines.

[116] Zhang H.-Q., Wang L.-J., Liu S.-H., Li J., Xiao L.-G., Yang G.-T. (2019). Adiponectin Regulates Bone Mass in AIS Osteopenia via RANKL/OPG and IL6 Pathway. J. Transl. Med..

[117] Sudan S.K., Deshmukh S.K., Poosarla T., Holliday N.P., Dyess D.L., Singh A.P., Singh S. (2020). Resistin: An Inflammatory Cytokine with Multi-Faceted Roles in Cancer. Biochim. Biophys. Acta Rev. Cancer.

[118] Muruganandan S., Ionescu A.M., Sinal C.J. (2020). At the Crossroads of the Adipocyte and Osteoclast Differentiation Programs: Future Therapeutic Perspectives. Int. J. Mol. Sci..

[119] Tzanavari T., Tasoulas J., Vakaki C., Mihailidou C., Tsourouflis G., Theocharis S. (2019). The Role of Adipokines in the Establishment and Progression of Head and Neck Neoplasms. Curr. Med. Chem..

[120] Chen S.-S., Tang C.-H., Chie M.-J., Tsai C.-H., Fong Y.-C., Lu Y.-C., Chen W.-C., Lai C.-T., Wei C.-Y., Tai H.-C. (2019). Resistin Facilitates VEGF-A-Dependent Angiogenesis by Inhibiting miR-16-5p in Human Chondrosarcoma Cells. Cell Death Dis..

[121] Lo J.C., Ljubicic S., Leibiger B., Kern M., Leibiger I.B., Moede T., Kelly M.E., Chatterjee Bhowmick D., Murano I., Cohen P. (2014). Adipsin Is an Adipokine That Improves β Cell Function in Diabetes. Cell.

[122] Liu Z., Xu J., He J., Liu H., Lin P., Wan X., Navone N.M., Tong Q., Kwak L.W., Orlowski R.Z. (2015). Mature Adipocytes in Bone Marrow Protect Myeloma Cells against Chemotherapy through Autophagy Activation. Oncotarget.

[123] Sheng X., Parmentier J.-H., Tucci J., Pei H., Cortez-Toledo O., Dieli-Conwright C.M., Oberley M.J., Neely M., Orgel E., Louie S.G. (2017). Adipocytes Sequester and Metabolize the Chemotherapeutic Daunorubicin. Mol. Cancer Res..

[124] Jones C.L., Stevens B.M., D’Alessandro A., Reisz J.A., Culp-Hill R., Nemkov T., Pei S., Khan N., Adane B., Ye H. (2019). Inhibition of Amino Acid Metabolism Selectively Targets Human Leukemia Stem Cells. Cancer Cell.

[125] Mah C.Y., Nassar Z.D., Swinnen J.V., Butler L.M. (2020). Lipogenic Effects of Androgen Signaling in Normal and Malignant Prostate. Asian J. Urol..

[126] Tabe Y., Yamamoto S., Saitoh K., Sekihara K., Monma N., Ikeo K., Mogushi K., Shikami M., Ruvolo V., Ishizawa J. (2017). Bone Marrow Adipocytes Facilitate Fatty Acid Oxidation Activating AMPK and a Transcriptional Network Supporting Survival of Acute Monocytic Leukemia Cells. Cancer Res..

[127] Foo B.J.-A., Eu J.Q., Hirpara J.L., Pervaiz S. (2021). Interplay between Mitochondrial Metabolism and Cellular Redox State Dictates Cancer Cell Survival. Oxid. Med. Cell. Longev..

[128] Lee I. (2021). Regulation of Cytochrome c Oxidase by Natural Compounds Resveratrol, (-)-Epicatechin, and Betaine. Cells.

[129] Hamabe-Horiike T., Harada S.-I., Yoshida K., Kinoshita J., Yamaguchi T., Fushida S. (2023). Adipocytes Contribute to Tumor Progression and Invasion of Peritoneal Metastasis by Interacting with Gastric Cancer Cells as Cancer Associated Fibroblasts. Cancer Rep..

[130] Chhabra Y., Weeraratna A.T. (2023). Fibroblasts in Cancer: Unity in Heterogeneity. Cell.

[131] Pang J., Shi Q., Liu Z., He J., Liu H., Lin P., Cui J., Yang J. (2017). Resistin Induces Multidrug Resistance in Myeloma by Inhibiting Cell Death and Upregulating ABC Transporter Expression. Haematologica.

[132] Lehuédé C., Li X., Dauvillier S., Vaysse C., Franchet C., Clement E., Esteve D., Longué M., Chaltiel L., Le Gonidec S. (2019). Adipocytes Promote Breast Cancer Resistance to Chemotherapy, a Process Amplified by Obesity: Role of the Major Vault Protein (MVP). Breast Cancer Res..

[133] Tuncer C., Hacioglu C. (2025). Notch1 and Major Vault Proteins Modulate Temozolomide Resistance in Glioblastoma. J. Cell. Mol. Med..

[134] Herroon M.K., Diedrich J.D., Rajagurubandara E., Martin C., Maddipati K.R., Kim S., Heath E.I., Granneman J., Podgorski I. (2019). Prostate Tumor Cell-Derived IL1β Induces an Inflammatory Phenotype in Bone Marrow Adipocytes and Reduces Sensitivity to Docetaxel via Lipolysis-Dependent Mechanisms. Mol. Cancer Res..

[135] Kumar B., Orellana M., Brooks J., Madabushi S.S., Vishwasrao P., Parra L.E., Sanchez J., Salhotra A., Stein A., Chen C.-C. (2020). Exosomes-Driven Lipolysis and Bone Marrow Niche Remodeling Supports Leukemia Expansion. Haematologica.

[136] Heuer T.S., Ventura R., Mordec K., Lai J., Fridlib M., Buckley D., Kemble G. (2017). FASN Inhibition and Taxane Treatment Combine to Enhance Anti-Tumor Efficacy in Diverse Xenograft Tumor Models through Disruption of Tubulin Palmitoylation and Microtubule Organization and FASN Inhibition-Mediated Effects on Oncogenic Signaling and Gene Expression. EBioMedicine.

[137] Fhu C.W., Ali A. (2020). Fatty Acid Synthase: An Emerging Target in Cancer. Molecules.

[138] Serhan H.A., Bao L., Cheng X., Qin Z., Liu C.-J., Heth J.A., Udager A.M., Soellner M.B., Merajver S.D., Morikawa A. (2024). Targeting Fatty Acid Synthase in Preclinical Models of TNBC Brain Metastases Synergizes with SN-38 and Impairs Invasion. NPJ Breast Cancer.

[139] Falchook G., Infante J., Arkenau H.-T., Patel M.R., Dean E., Borazanci E., Brenner A., Cook N., Lopez J., Pant S. (2021). First-in-Human Study of the Safety, Pharmacokinetics, and Pharmacodynamics of First-in-Class Fatty Acid Synthase Inhibitor TVB-2640 Alone and with a Taxane in Advanced Tumors. EClinicalMedicine.

[140] Xu J., Chen C., Sun K., Shi Q., Wang B., Huang Y., Ren T., Tang X. (2023). Tocilizumab (Monoclonal Anti-IL-6R Antibody) Reverses Anlotinib Resistance in Osteosarcoma. Front. Oncol..

[141] Yang H., Karl M.N., Wang W., Starich B., Tan H., Kiemen A., Pucsek A.B., Kuo Y.-H., Russo G.C., Pan T. (2022). Engineered Bispecific Antibodies Targeting the Interleukin-6 and -8 Receptors Potently Inhibit Cancer Cell Migration and Tumor Metastasis. Mol. Ther..

[142] Hardaway A.L., Herroon M.K., Rajagurubandara E., Podgorski I. (2015). Marrow Adipocyte-Derived CXCL1 and CXCL2 Contribute to Osteolysis in Metastatic Prostate Cancer. Clin. Exp. Metastasis.

[143] Yang Y., Luo X., Yan F., Jiang Z., Li Y., Fang C., Shen J. (2015). Effect of Zoledronic Acid on Vertebral Marrow Adiposity in Postmenopausal Osteoporosis Assessed by MR Spectroscopy. Skeletal Radiol..

[144] Li G.-W., Chang S.-X., Fan J.-Z., Tian Y.-N., Xu Z., He Y.-M. (2013). Marrow Adiposity Recovery after Early Zoledronic Acid Treatment of Glucocorticoid-Induced Bone Loss in Rabbits Assessed by Magnetic Resonance Spectroscopy. Bone.

[145] Smith M.R., Saad F., Coleman R., Shore N., Fizazi K., Tombal B., Miller K., Sieber P., Karsh L., Damião R. (2012). Denosumab and Bone-Metastasis-Free Survival in Men with Castration-Resistant Prostate Cancer: Results of a Phase 3, Randomised, Placebo-Controlled Trial. Lancet.

[146] Coleman R., Finkelstein D.M., Barrios C., Martin M., Iwata H., Hegg R., Glaspy J., Periañez A.M., Tonkin K., Deleu I. (2020). Adjuvant Denosumab in Early Breast Cancer (D-CARE): An International, Multicentre, Randomised, Controlled, Phase 3 Trial. Lancet Oncol..

[147] Bessot A., Gunter J., McGovern J., Bock N. (2025). Bone Marrow Adipocytes in Cancer: Mechanisms, Models, and Therapeutic Implications. Biomaterials.

[148] Hardaway A.L., Herroon M.K., Rajagurubandara E., Podgorski I. (2014). Bone Marrow Fat: Linking Adipocyte-Induced Inflammation with Skeletal Metastases. Cancer Metastasis Rev..

[149] Reagan M.R., Rosen C.J. (2016). Navigating the Bone Marrow Niche: Translational Insights and Cancer-Driven Dysfunction. Nat. Rev. Rheumatol..

[150] Fairfield H., Falank C., Farrell M., Vary C., Boucher J.M., Driscoll H., Liaw L., Rosen C.J., Reagan M.R. (2019). Development of a 3D Bone Marrow Adipose Tissue Model. Bone.

[151] Ottewell P.D., O’Donnell L., Holen I. (2015). Molecular Alterations That Drive Breast Cancer Metastasis to Bone. BoneKEy Rep..

[152] Lecka-Czernik B., Stechschulte L.A. (2014). Bone and Fat: A Relationship of Different Shades. Arch. Biochem. Biophys..

[153] Liu Y., Wu W., Cai C., Zhang H., Shen H., Han Y. (2023). Patient-Derived Xenograft Models in Cancer Therapy: Technologies and Applications. Signal Transduct. Target. Ther..

[154] Morris E.V., Edwards C.M. (2016). The Role of Bone Marrow Adipocytes in Bone Metastasis. J. Bone Oncol..

[155] Karampinos D.C., Ruschke S., Dieckmeyer M., Diefenbach M., Franz D., Gersing A.S., Krug R., Baum T. (2018). Quantitative MRI and Spectroscopy of Bone Marrow. Magn. Reson. Imaging.

[156] Agazzi G.M., Di Meo N., Rondi P., Saeli C., Dalla Volta A., Vezzoli M., Berruti A., Borghesi A., Maroldi R., Ravanelli M. (2024). Fat Fraction Extracted from Whole-Body Magnetic Resonance (WB-MR) in Bone Metastatic Prostate Cancer: Intra- and Inter-Reader Agreement of Single-Slice and Volumetric Measurements. Tomography.

[157] Faubert B., Tasdogan A. (2025). Approaches to Stable Isotope Tracing and in Vivo Metabolomics in the Cancer Clinic. EMBO J..

[158] Cristini N., Tavakoli M., Sanati M., Yavari S.A. (2025). Exploring Bone-Tumor Interactions through 3D in Vitro Models: Implications for Primary and Metastatic Cancers. J. Bone Oncol..

[159] Morris E.V., Edwards C.M. (2016). Bone Marrow Adipose Tissue: A New Player in Cancer Metastasis to Bone. Front. Endocrinol..

[160] Otley M.O.C., Sinal C.J. (2022). Adipocyte-Cancer Cell Interactions in the Bone Microenvironment. Front. Endocrinol..

[161] Cao J., Li Q., Zhang H., Wu Y., Wang X., Ding S., Chen S., Xu S., Duan G., Qiu D. (2024). Radiomics Model Based on MRI to Differentiate Spinal Multiple Myeloma from Metastases: A Two-Center Study. J. Bone Oncol..

[162] Veldhuis-Vlug A.G., Rosen C.J. (2018). Clinical Implications of Bone Marrow Adiposity. J. Intern. Med..

[163] Schwartz A.V. (2015). Marrow Fat and Bone: Review of Clinical Findings. Front. Endocrinol..

[164] Hardouin P., Pansini V., Cortet B. (2014). Bone Marrow Fat. Jt. Bone Spine.

[165] El-Masri B.M., Leka B., Mustapha F., Gundesen M.T., Hinge M., Lund T., Andersen T.L., Diaz-delCastillo M., Jafari A. (2024). Bone Marrow Adipocytes Provide Early Sign for Progression from MGUS to Multiple Myeloma. Oncotarget.

[166] Zhang F., Guo J., Zhang Z., Qian Y., Wang G., Duan M., Zhao H., Yang Z., Jiang X. (2022). Mesenchymal Stem Cell-Derived Exosome: A Tumor Regulator and Carrier for Targeted Tumor Therapy. Cancer Lett..

[167] Awad D., Cao P.H.A., Pulliam T.L., Spradlin M., Subramani E., Tellman T.V., Ribeiro C.F., Muzzioli R., Jewell B.E., Pakula H. (2024). Adipose Triglyceride Lipase Is a Therapeutic Target in Advanced Prostate Cancer That Promotes Metabolic Plasticity. Cancer Res..

[168] Mayer N., Schweiger M., Romauch M., Grabner G.F., Eichmann T.O., Fuchs E., Ivkovic J., Heier C., Mrak I., Lass A. (2013). Development of Small-Molecule Inhibitors Targeting Adipose Triglyceride Lipase. Nat. Chem. Biol..

[169] Iglesias J., Lamontagne J., Erb H., Gezzar S., Zhao S., Joly E., Truong V.L., Skorey K., Crane S., Madiraju S.R.M. (2016). Simplified Assays of Lipolysis Enzymes for Drug Discovery and Specificity Assessment of Known Inhibitors. J. Lipid Res..

[170] Schweiger M., Romauch M., Schreiber R., Grabner G.F., Hütter S., Kotzbeck P., Benedikt P., Eichmann T.O., Yamada S., Knittelfelder O. (2017). Pharmacological Inhibition of Adipose Triglyceride Lipase Corrects High-Fat Diet-Induced Insulin Resistance and Hepatosteatosis in Mice. Nat. Commun..

[171] Xie H., Heier C., Kien B., Vesely P.W., Tang Z., Sexl V., Schoiswohl G., Strießnig-Bina I., Hoefler G., Zechner R. (2020). Adipose Triglyceride Lipase Activity Regulates Cancer Cell Proliferation via AMP-Kinase and mTOR Signaling. Biochim. Biophys. Acta Mol. Cell Biol. Lipids.

[172] Koh Y.J., Park B.-H., Park J.-H., Han J., Lee I.-K., Park J.W., Koh G.Y. (2009). Activation of PPAR Gamma Induces Profound Multilocularization of Adipocytes in Adult Mouse White Adipose Tissues. Exp. Mol. Med..

[173] Kraakman M.J., Liu Q., Postigo-Fernandez J., Ji R., Kon N., Larrea D., Namwanje M., Fan L., Chan M., Area-Gomez E. (2018). PPARγ Deacetylation Dissociates Thiazolidinedione’s Metabolic Benefits from Its Adverse Effects. J. Clin. Investig..

[174] Seki T., Yang Y., Sun X., Lim S., Xie S., Guo Z., Xiong W., Kuroda M., Sakaue H., Hosaka K. (2022). Brown-Fat-Mediated Tumour Suppression by Cold-Altered Global Metabolism. Nature.

[175] Chen Z., Kang Y. (2023). Cold Snap for Cancer: Cold-Induced Brown Fat Thermogenesis Starves Tumor Growth. Signal Transduct. Target. Ther..

[176] Blake M.L., Tometsko M., Miller R., Jones J.C., Dougall W.C. (2014). RANK Expression on Breast Cancer Cells Promotes Skeletal Metastasis. Clin. Exp. Metastasis.

[177] Jones D.H., Nakashima T., Sanchez O.H., Kozieradzki I., Komarova S.V., Sarosi I., Morony S., Rubin E., Sarao R., Hojilla C.V. (2006). Regulation of Cancer Cell Migration and Bone Metastasis by RANKL. Nature.

[178] Tang Z.-N., Zhang F., Tang P., Qi X.-W., Jiang J. (2011). Hypoxia Induces RANK and RANKL Expression by Activating HIF-1α in Breast Cancer Cells. Biochem. Biophys. Res. Commun..

[179] Vargas G., Bouchet M., Bouazza L., Reboul P., Boyault C., Gervais M., Kan C., Benetollo C., Brevet M., Croset M. (2019). ERRα Promotes Breast Cancer Cell Dissemination to Bone by Increasing RANK Expression in Primary Breast Tumors. Oncogene.

[180] Onji M., Werschler N., Penninger J. (2021). A Critical Relationship between Bone and Fat: The Role of Bone Marrow Adipose-Derived RANKL in Bone Metabolism. EMBO Rep..

[181] Beekman K.M., Zwaagstra M., Veldhuis-Vlug A.G., van Essen H.W., den Heijer M., Maas M., Kerckhofs G., Parac-Vogt T.N., Bisschop P.H., Bravenboer N. (2019). Ovariectomy Increases RANKL Protein Expression in Bone Marrow Adipocytes of C3H/HeJ Mice. Am. J. Physiol. Endocrinol. Metab..

[182] Méndez-Clemente A., Bravo-Cuellar A., González-Ochoa S., Santiago-Mercado M., Palafox-Mariscal L., Jave-Suárez L., Solorzano-Ibarra F., Villaseñor-García M., Ortiz-Lazareno P., Hernández-Flores G. (2022). Dual STAT-3 and IL-6R Inhibition with Stattic and Tocilizumab Decreases Migration, Invasion and Proliferation of Prostate Cancer Cells by Targeting the IL-6/IL-6R/STAT-3 Axis. Oncol. Rep..

[183] Ali D., Tencerova M., Figeac F., Kassem M., Jafari A. (2022). The Pathophysiology of Osteoporosis in Obesity and Type 2 Diabetes in Aging Women and Men: The Mechanisms and Roles of Increased Bone Marrow Adiposity. Front. Endocrinol..

[184] Kim T.Y., Schafer A.L. (2016). Diabetes and Bone Marrow Adiposity. Curr. Osteoporos. Rep..

[185] Chen K., Li X., Chen D., Qian S., Mei R., Li Q., Yu X., He X. (2025). PPARγ Inhibitors Enhance the Efficacy of Statin Therapy for Steroid-Induced Osteonecrosis of the Femoral Head by Directly Inhibiting Apoptosis and Indirectly Modulating Lipoprotein Subfractions. PLoS ONE.

[186] McGrath C., Sankaran J.S., Misaghian-Xanthos N., Sen B., Xie Z., Styner M.A., Zong X., Rubin J., Styner M. (2019). Exercise Degrades Bone in Caloric Restriction, Despite Suppression of Marrow Adipose Tissue (MAT). J. Bone Miner. Res..

[187] Rinne C., Soultoukis G.A., Oveisi M., Leer M., Schmidt-Bleek O., Burkhardt L.-M., Bucher C.H., Moussa E.A., Makhlouf M., Duda G.N. (2024). Caloric Restriction Reduces Trabecular Bone Loss during Aging and Improves Bone Marrow Adipocyte Endocrine Function in Male Mice. Front. Endocrinol..

[188] Lin W., Li Y., Qiu C., Zou B., Gong Y., Zhang X., Tian D., Sherman W., Sanchez F., Wu D. (2025). Mapping the Spatial Atlas of the Human Bone Tissue Integrating Spatial and Single-Cell Transcriptomics. Nucleic Acids Res..

[189] Bandyopadhyay S., Duffy M.P., Ahn K.J., Sussman J.H., Pang M., Smith D., Duncan G., Zhang I., Huang J., Lin Y. (2024). Mapping the Cellular Biogeography of Human Bone Marrow Niches Using Single-Cell Transcriptomics and Proteomic Imaging. Cell.

[190] Baccin C., Al-Sabah J., Velten L., Helbling P.M., Grünschläger F., Hernández-Malmierca P., Nombela-Arrieta C., Steinmetz L.M., Trumpp A., Haas S. (2020). Combined Single-Cell and Spatial Transcriptomics Reveal the Molecular, Cellular and Spatial Bone Marrow Niche Organization. Nat. Cell Biol..

[191] Faubert B., Tasdogan A., Morrison S.J., Mathews T.P., DeBerardinis R.J. (2021). Stable Isotope Tracing to Assess Tumor Metabolism in Vivo. Nat. Protoc..

[192] Kim I.-Y., Park S., Jang J., Wolfe R.R. (2020). Quantifications of Lipid Kinetics In Vivo Using Stable Isotope Tracer Methodology. J. Lipid Atheroscler..

[193] Miggitsch C., Meryk A., Naismith E., Pangrazzi L., Ejaz A., Jenewein B., Wagner S., Nägele F., Fenkart G., Trieb K. (2019). Human Bone Marrow Adipocytes Display Distinct Immune Regulatory Properties. EBioMedicine.

[194] Hu T., Liu C.-H., Lei M., Zeng Q., Li L., Tang H., Zhang N. (2024). Metabolic Regulation of the Immune System in Health and Diseases: Mechanisms and Interventions. Signal Transduct. Target. Ther..

[195] Philippoteaux C., Badr S., Lombardo D., Cailliau E., Ruschke S., Karampinos D.C., Cotten A., Paccou J. (2025). Marrow Adiposity Content and Composition Are Not Associated With Incident Fragility Fractures in Postmenopausal Women: The ADIMOS Fracture Study. J. Endocr. Soc..

[196] Idilman I.S., Yildiz A.E., Karaosmanoglu A.D., Ozmen M.N., Akata D., Karcaaltincaba M. (2022). Proton Density Fat Fraction: Magnetic Resonance Imaging Applications beyond the Liver. Diagn. Interv. Radiol..

[197] Dembitz V., James S.C., Gallipoli P. (2025). Targeting Lipid Metabolism in Acute Myeloid Leukemia: Biological Insights and Therapeutic Opportunities. Leukemia.

[198] Samudio I., Harmancey R., Fiegl M., Kantarjian H., Konopleva M., Korchin B., Kaluarachchi K., Bornmann W., Duvvuri S., Taegtmeyer H. (2010). Pharmacologic Inhibition of Fatty Acid Oxidation Sensitizes Human Leukemia Cells to Apoptosis Induction. J. Clin. Investig..

[199] Raje N., Terpos E., Willenbacher W., Shimizu K., García-Sanz R., Durie B., Legieć W., Krejčí M., Laribi K., Zhu L. (2018). Denosumab versus Zoledronic Acid in Bone Disease Treatment of Newly Diagnosed Multiple Myeloma: An International, Double-Blind, Double-Dummy, Randomised, Controlled, Phase 3 Study. Lancet Oncol..

[200] Beekman K.M., Regenboog M., Nederveen A.J., Bravenboer N., den Heijer M., Bisschop P.H., Hollak C.E., Akkerman E.M., Maas M. (2022). Gender- and Age-Associated Differences in Bone Marrow Adipose Tissue and Bone Marrow Fat Unsaturation Throughout the Skeleton, Quantified Using Chemical Shift Encoding-Based Water-Fat MRI. Front. Endocrinol..

[201] Serota A., D’Erasmo G. (2024). Estrogen Exposure and Skeletal Health: Special Populations and Considerations. J. Pediatr. Soc. N. Am..

